# The Complex I Subunit *NDUFA10* Selectively Rescues *Drosophila pink1* Mutants through a Mechanism Independent of Mitophagy

**DOI:** 10.1371/journal.pgen.1004815

**Published:** 2014-11-20

**Authors:** Joe H. Pogson, Rachael M. Ivatt, Alvaro Sanchez-Martinez, Roberta Tufi, Emma Wilson, Heather Mortiboys, Alexander J. Whitworth

**Affiliations:** 1 MRC Centre for Developmental and Biomedical Genetics, Sheffield, United Kingdom; 2 Department of Biomedical Sciences, University of Sheffield, Sheffield, United Kingdom; 3 Sheffield Institute for Translational Neuroscience, University of Sheffield, Sheffield, United Kingdom; Stanford University School of Medicine, United States of America

## Abstract

Mutations in PINK1, a mitochondrially targeted serine/threonine kinase, cause autosomal recessive Parkinson's disease (PD). Substantial evidence indicates that PINK1 acts with another PD gene, *parkin*, to regulate mitochondrial morphology and mitophagy. However, loss of PINK1 also causes complex I (CI) deficiency, and has recently been suggested to regulate CI through phosphorylation of NDUFA10/ND42 subunit. To further explore the mechanisms by which PINK1 and Parkin influence mitochondrial integrity, we conducted a screen in *Drosophila* cells for genes that either phenocopy or suppress mitochondrial hyperfusion caused by *pink1* RNAi. Among the genes recovered from this screen was *ND42*. In *Drosophila pink1* mutants, transgenic overexpression of *ND42* or its co-chaperone *sicily* was sufficient to restore CI activity and partially rescue several phenotypes including flight and climbing deficits and mitochondrial disruption in flight muscles. Here, the restoration of CI activity and partial rescue of locomotion does not appear to have a specific requirement for phosphorylation of ND42 at Ser-250. In contrast to *pink1* mutants, overexpression of *ND42* or *sicily* failed to rescue any *Drosophila parkin* mutant phenotypes. We also find that knockdown of the human homologue, *NDUFA10*, only minimally affecting CCCP-induced mitophagy, and overexpression of *NDUFA10* fails to restore Parkin mitochondrial-translocation upon *PINK1* loss. These results indicate that the *in vivo* rescue is due to restoring CI activity rather than promoting mitophagy. Our findings support the emerging view that PINK1 plays a role in regulating CI activity separate from its role with Parkin in mitophagy.

## Author Summary

Two genes linked to heritable forms of the neurodegenerative movement disorder Parkinson's disease (PD), *PINK1* and *parkin*, play important roles in mitochondrial homeostasis through mechanisms which include the degradation of dysfunctional mitochondria, termed mitophagy, and the maintenance of complex I (CI) activity. Here we report the findings of an RNAi based screen in *Drosophila* cells for genes that may regulate the PINK1-Parkin pathway which identified *NDUFA10* (*ND42* in *Drosophila*), a subunit of CI. Using a well-established cellular system and *in vivo Drosophila* genetics, we demonstrate that while NDUFA10/ND42 only plays a minimal role in mitophagy, restoration of CI activity through overexpression of either *ND42* or its co-chaperone *sicily* is able to substantially rescue behavioral deficits in *pink1* mutants but not *parkin* mutants. Moreover, while *parkin* overexpression is known to rescue *pink1* mutants, it apparently achieves this without restoring CI activity. These results suggest that increasing CI activity or promoting mitophagy can be beneficial in *pink1* mutants, and further highlights separable functions of PINK1 and Parkin.

## Introduction

Parkinson's disease (PD) is the second most prevalent neurodegenerative disorder, the etiology of which remains unknown. Mitochondrial dysfunction, including complex I (CI) deficiency, are frequently observed in pathologic specimens. To elucidate the underlying molecular events, intensive research has focused on identifying the causative gene mutations for inherited forms of PD. An impressive array of disease-causing mutations have been found including autosomal recessive mutations in *PARK6* which encodes PTEN-Induced Kinase 1 (PINK1), a mitochondrially targeted serine/threonine kinase, and *PARK2*, encoding the cytoplasmic E3 ubiquitin ligase Parkin [1], [2]. Previous work has shown that PINK1 and Parkin regulate several cellular processes that impinge on mitochondrial homeostasis [3]–[13], including the fission-fusion dynamics and trafficking of mitochondria [14]–[17], the degradation of damaged mitochondria [18]–[22], inter-organelle communication [23], and activity of CI of the electron transport chain [4], [8], [9], [24], [25]. However, the mechanisms by which PINK1 and Parkin affect these processes is incompletely understood.

In order to identify functional partners of PINK1 we performed a cell based RNAi screen to identify genes that either phenocopy or suppress *PINK1* phenotypes. In *Drosophila* cells lacking *pink1* or *parkin* steady state levels of Marf are increased causing excess fusion of the mitochondrial network [14], [17], [26]. This imbalance in mitochondrial fission-fusion at least partially contributes to the observed organismal phenotypes – enlarged and disrupted mitochondria, muscle degeneration, male sterility, and associated behavioral deficits and neurodegeneration – since they can be partially rescued by genetic interaction with fission (*Drp1*) or fusion (*OPA1* and *Marf*) genes to restore the balance of normal mitochondrial morphology [14], [16]. We used this phenotype to screen for factors that impact on mitochondrial morphology and which may play a role in PINK1/Parkin function. Our RNAi screen identified multiple hits including a subunit of CI, ND42/NDUFA10. Given the link between CI deficiency and PD, we selected ND42/NDUFA10 for further characterization.

Knockdown of *Drosophila ND42* phenocopied *pink1* RNAi, causing excessive mitochondrial fusion. Genetic studies in *Drosophila* reveal that overexpression of *ND42* or its co-chaperone *sicily* is sufficient to rescue behavioral deficits in *pink1* mutants through restoration of CI activity. In contrast, overexpression of neither *ND42* nor *sicily* rescued *parkin* mutant flies, and attenuation of the mammalian homolog, *NDUFA10*, in HeLa cells only modestly reduced mitophagy. Furthermore, *NDUFA10* overexpression cannot restore Parkin translocation to mitochondria in the absence of PINK1, suggesting that ND42 selectively rescues *pink1* mutants through a mechanism independent of mitophagy. Our study provides additional evidence in support of a role for PINK1 in CI activity, and further highlights separable functions of PINK1 and Parkin. Future characterization of the other factors from our screen promises to shed additional light on the functional roles of PINK1 and Parkin.

## Results

### An RNAi screen for suppressors or phenocopiers of *pink1* RNAi-induced mitochondrial fusion

**Figure 1:**
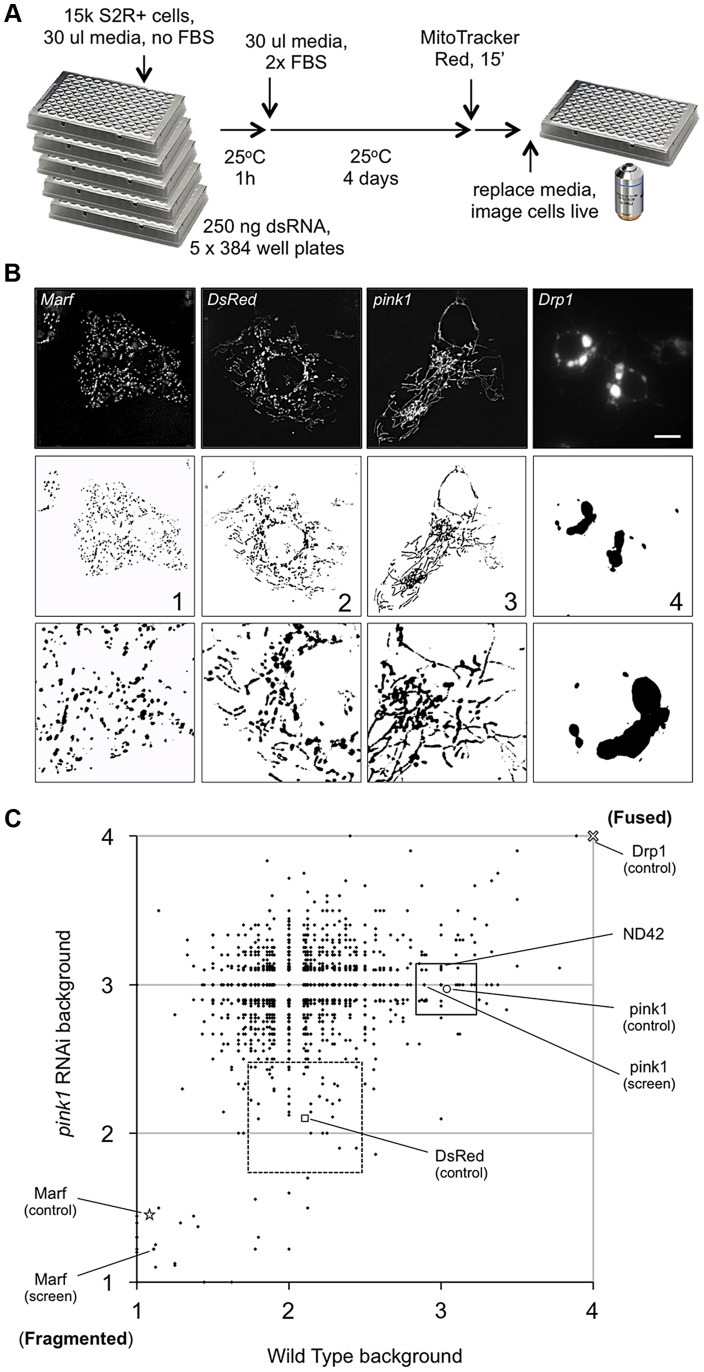
A cell based RNAi screen to identify phenocopiers and suppressors of *pink1* RNAi-induced mitochondrial hyperfusion. (A) Schematic of the RNAi screen protocol (see Methods for details). (B) Representative images of *Drosophila* S2R+ cells for mitochondrial morphology following dsRNA treatment of the indicated genes, stained with MitoTracker Red and imaged live (top row). Fluorescence images are converted to binary (B&W) and inverted to clarify the mitochondrial morphology (bottom row). Numbers represent the designated ‘morphology score’: 1, fragmented; 2, wild type; 3, fused/tubular; 4, hyperfused/clumped. (C) Comparison of morphology score of screen library amplicons in WT and *pink1* RNAi backgrounds. Solid-line box depicts those amplicons that phenocopy *pink1* RNAi (box limits: mean ± s.d. *pink1* control). Dashed-line box depicts amplicons which suppress *pink1* RNAi-induced fusion back to WT morphology (box limits: mean ± s.d. *DsRed* control). Controls for fragmentation (*Marf)* and fusion (*Drp1*) are shown. Scale bar  = 10 µm.

To identify new components in PINK1-Parkin pathway we performed an RNAi screen in *Drosophila* cells, using a subset library enriched for kinases and phosphatases, for genes that alter mitochondrial morphology (Figure 1A). In particular, we sought to identify genes whose knockdown could either phenocopy or suppress the *pink1* RNAi-induced excess fusion. dsRNA treated cells were imaged live using MitoTracker Red to fluorescently label the mitochondrial network. The mitochondrial morphology in each image was scored as primarily falling into one of four categories based on control knockdown phenotypes (Figure 1B). Control (*DsRed*) dsRNA treated cells showed a typical mix of short-round and long-tubular mitochondria. This category was scored 2. Cells treated with dsRNA against the fly mitofusin homologue, *Marf*, caused a complete fragmentation of the mitochondrial network as expected and were scored 1. *pink1* RNAi resulted in a tubular, highly interconnected network, as previously reported [14], [17], scoring 3. RNAi against the pro-fission factor *Drp1* caused mitochondrial hyperfusion and peri-nuclear clumping. Being qualitatively different from the *pink1* RNAi phenotype and an extreme result of hyperfusion this category scored 4.

The screen was performed on a library of selected genes mostly comprising kinases and phosphatases but with additional genes of interest. The effect on mitochondrial morphology was assessed in two backgrounds; one in a wild type background to identify manipulations that phenocopy *pink1* RNAi tubulation, and another in a *pink1* RNAi background to identify manipulations capable of suppressing the *pink1* phenotype. The results from the two screens were cross-referenced to further refine the selection of possible hits (Figure 1C). The limits of the regions considered to phenocopy or suppress were defined by the mean ± standard deviation for *pink1* or *DsRed* dsRNAs respectively. The gene targets whose dsRNAs either phenocopy or suppress *pink1* RNAi are summarized in Table 1 and Table 2.

**Table 1:** List of genes that phenocopy *pink1* dsRNA induced mitochondrial fusion.

Phenocopiers				
CG#	Gene name	Molecular Function	Human Homologue	Accession #
CG4523	pink1	Serine/Threonine Kinase	PTEN–induced kinase 1	NP_115785
CG2277	CG2277	Unknown	5'–nucleotidase domain–containing protein 1	NP_689942
CG32505	Pp4–19C	Serine/Threonine Phosphatase	serine/threonine–protein phosphatase 4 catalytic subunit	NP_002711
CG42341	Pka–R1	cAMP Dependent Kinase	cAMP–dependent protein kinase type I–beta regulatory subunit	NP_002726
CG10564	Ac78c	Adenylate Cyclase Activity	Adenylate cyclase type 8	NP_001106
CG10261	aPkc	Serine/Threonine Kinase	protein kinase C iota type	NP_002731
CG7004	Four wheel drive	Phosphatidylinositol 4–Kinase	phosphatidylinositol 4–kinase beta isoform 2	NP_001185703
CG11870	CG11870	Protein Kinase	NUAK Family SNF1–like kinase	NP_055655
CG1747	Sk1	Sphingosine Kinase	Sphingosine Kinase 1	NP_068807
CG6343	ND42	NADH Dehydrogenase	NDUFA10	NP_004535
CG34412	Tousled like kinase	Protein Kinase	serine/threonine–protein kinase tousled–like 2	NP_036033
CG42317	Csk	Tyrosine Kinase	tyrosine–protein kinase C-terminal Src Kinase	NP_004374

**Table 2:** List of genes that suppress *pink1* dsRNA induced mitochondrial fusion.

Suppressors				
CG#	Gene name	Molecular Function	Human Homologue	Accession #
CG5656	CG5656	Alkaline phosphatase	Alkaline Phosphatase	NP_001170991.1
CG8128	CG8128	Nudix hydrolase activity	uridine diphosphate glucose pyrophosphatase	NP_803877
CG33991	nuclear fallout	Rab and microtubule binding	Rab11 FIP3/Rab11 FIP4	NP_055515.1/NP_116321.2
CG4266	CG4266	mRNA binding	RBM16	NP_055707.3
CG7899	Acph–1	Acid Phosphatase	prostatic acid phosphatase isoform TM–PAP precursor	NP_001127666.1
CG7431	CG7431	Tyramine Receptor	Alpha–1b adrenergic receptor	NP_000670
CG31759	CG31759	unknown	2′5′–phophodiesterase 12	NP_808881
CG17559	doughnut on 2	Receptor	tyrosine–protein kinase RYK isoform 2 precursor	NP_002949
CG10975	Ptp69D	Tyrosine Phosphatase	receptor–type tyrosine–protein phosphatase alpha isoform 2 precursor	NP_543030
CG5361	CG5361	Alkaline Phosphatase	alkaline phosphatase, tissue–nonspecific isozyme isoform 1 precursor	NP_000469
CG3980	Cep97	Protein Phosphatase	Cep97	NP_078824
CG3525	easily shocked	Ethanolamine Kinase	Ethanolamine kinase 1	NP_061108.2
CG3494	CG3494	unknown	Leucine rich repeat containing protein 40	NP_060238
CG11660	CG11660	Protein Kinase	Serine/threonine kinase RIO1	NP_113668.2
CG1637	CG1637	Acid Phosphatase	Iron/Zinc purple acid phosphatase	NP_001004318.2
CG40448	Pp1–Y2	Serine/Threonine Phosphatase	Serine/threonine phosphatase PP1 gamma	NP_002701
CG17746	CG17746	Serine/Threonine Phosphatase	Protein phosphatase 1A	NP_808820
CG7180	CG7180	Tyrosine Phosphatase	receptor–type tyrosine–protein phosphatase kappa isoform b precursor	NP_002835
CG31299	curled	Unknown	nocturnin	NP_036250
CG6860	Lrch	Unknown	Leucine rich repeat and calponin homology	NP_116162
CG3051	SNF1A	AMP–activated protein kinase	5'–AMP–activated protein kinase catalytic subunit alpha–2	NP_006243
CG34356	CG34356	Protein Kinase	SCY1–like protein 2	NP_060458
CG42366	CG42366	Protein Kinase	Serine/Threonine Kinase ICK	NP_057597
CG3530	CG3530	Tyrosine/Serine/Threonine kinase	Myotubularin	NP_000243

While there are several interesting candidates for further investigation in both suppressors and phenocopiers (see Discussion), a specific motivation for this work was to identify additional components of the PINK1-Parkin pathway that regulate mitochondrial dynamics and mitophagy. An attractive candidate that could fulfill this role would be a mitochondrially localized protein. Notably, we identified *ND42*, which encodes a subunit of CI of the electron transport chain, as a phenocopier; knockdown of *ND42* caused excess tubulation similar to *pink1* knockdown. Importantly, CI activity has been shown to be specifically affected in various models of *pink1* deficiency, supporting ND42 as an attractive target for analysis in pink1 function.

### *ND42* knockdown specifically phenocopies *pink1* RNAi induced mitochondrial hyperfusion

CI is a very large, multi-subunit complex comprising of around 44 subunits, consisting of a hydrophobic portion embedded in the inner membrane and a hydrophilic portion extending into the matrix [27], [28]. In order to assess the specific versus general effect of attenuating CI subunits on mitochondrial morphology, several additional subunits were analyzed. dsRNAs targeting 6 other subunits from different subdomains (α, β or λ) were generated and the effect of knockdown on mitochondrial morphology was assessed.

**Figure 2:**
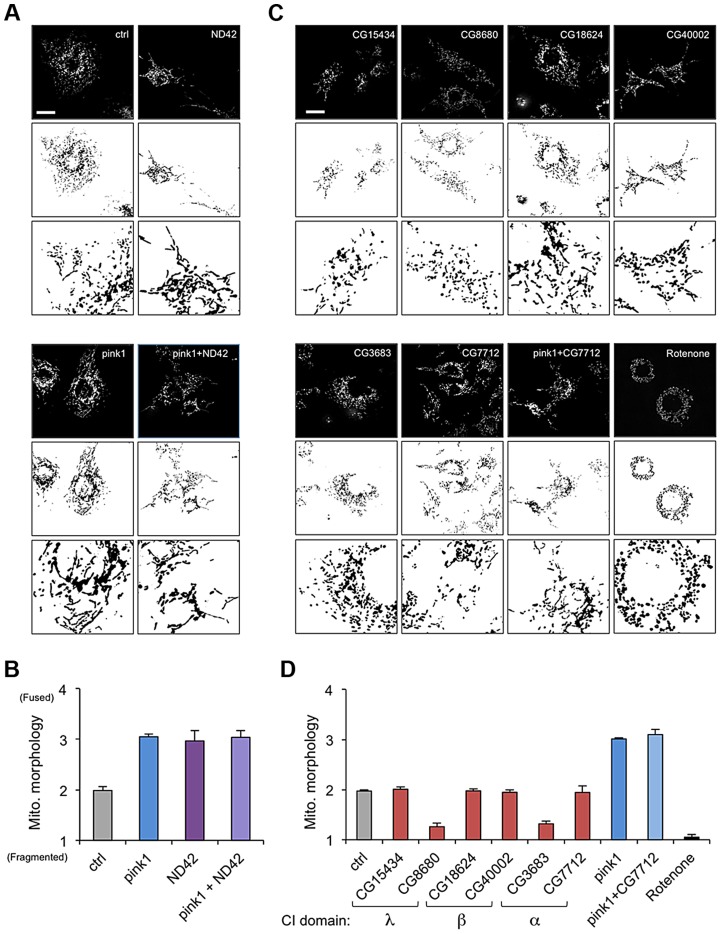
*ND42* RNAi but not other complex I subunits phenocopies *pink1* RNAi. (A) *ND42* RNAi in *Drosophila* S2R+ cells stained with MitoTracker Red causes tubulation of the mitochondrial network, similar to *pink1* RNAi. *ND42* RNAi does not further perturb morphology in conjunction with *pink1* RNAi. (B) Quantification of mitochondrial morphology as in A, scored in triplicate experiments. (C) RNAi of selected subunits of complex I or rotenone treatment do not phenocopy *pink1* RNAi. (D) Quantification of morphology scored in triplicate experiments as in C. Histograms indicate mean ± s.d. of triplicate experiments. Inverted, binary images are shown below each fluorescence image to aid clarity of mitochondrial morphology. n>30 cells per experiment. Scale bar  = 10 µm.

We confirmed that *ND42* knockdown caused excess mitochondrial fusion in a WT background, indistinguishable from *pink1* knockdown, but did not further enhance the *pink1* phenotype (Figures 2A, 2B, and S1). In contrast none of the other selected subunits induced fusion; 4 subunits had no effect on morphology while 2 subunits caused fragmentation (Figures 2C and 2D). Further analysis of one of these subunits, CG7712, which did not perturb mitochondrial morphology, also did not modify the *pink1* phenotype. These results support the specificity of the effect observed with *ND42* knockdown.

### *ND42* overexpression can rescue *pink1* but not *parkin* mutants

Since *ND42* RNAi phenocopies loss of *pink1* in cells we assessed whether loss of *ND42* may phenocopy *pink1* mutants *in vivo*. *Drosophila* mutant for *pink1* exhibit characteristic locomotor deficits in climbing and flight, associated with degeneration of the musculature and profound disruption of mitochondria [3], [11]. In agreement with previous observations [29], we found that knockdown of *ND42* in all tissues is lethal, consistent with the subunit playing a critical role in this essential metabolic enzyme.

**Figure 3:**
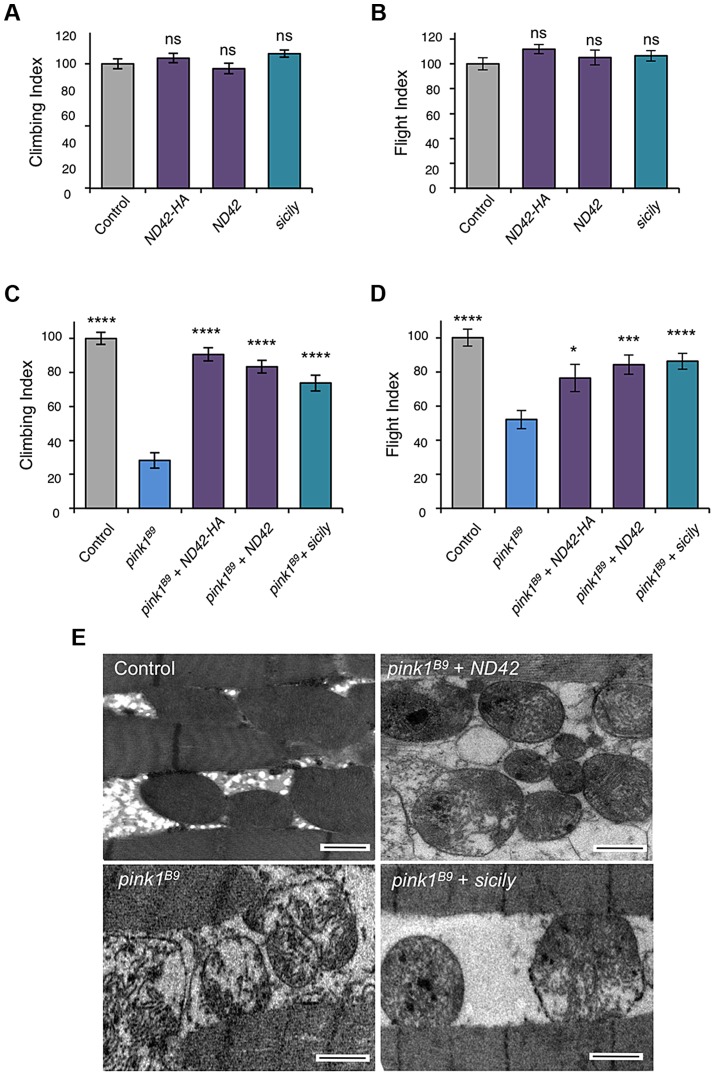
*ND42* or *sicily* overexpression can rescue *pink1* mutant phenotypes in flies. Overexpression of two *ND42* transgenes, *ND42* and *ND42-HA*, in a wild type background has no effect on climbing (A) or flight behavior (B). In *pink1^B9^* mutants, climbing (C) and flight ability (D), normalized to control, is significantly rescued by overexpression *ND42* or *sicily*. Histograms indicate mean ± s.e.m. (E) Transmission electron microscopy of flight muscle shows partial rescue of mitochondrial disruption. Scale bar  = 1 µm. Overexpression was driven by the ubiquitous driver *da-GAL4*. Control genotype is *da-GAL4*/+. Number of animals tested, n>50. * *P*<0.05, *** *P*<0.001, **** *P*<0.0001, One-way ANOVA with Bonferroni correction. Comparisons are with control (A, B) or *pink1^B9^* mutants (C, D).

Due to the essential nature of this gene, and lack of *pink1* phenocopy, we did not further characterize *ND42* loss-of-function. We hypothesized that, since *pink1* mutants show CI deficiency, overexpressing *ND42* may suppress *pink1* phenotypes. While *ND42* overexpression of two independent transgenes driven by the strong ubiquitous driver *daughterless* (*da*)-*GAL4* had no effect on motor performance in a wild type background (Figures 3A and 3B), we found that expression of either transgene was able to significantly restore climbing and flight ability in *pink1* mutants (Figures 3C and 3D). However, *ND42* overexpression only partially restored flight muscle and mitochondrial integrity (Figure 3E), but was not able to improve the male sterility (Figure S2A).

**Figure 4:**
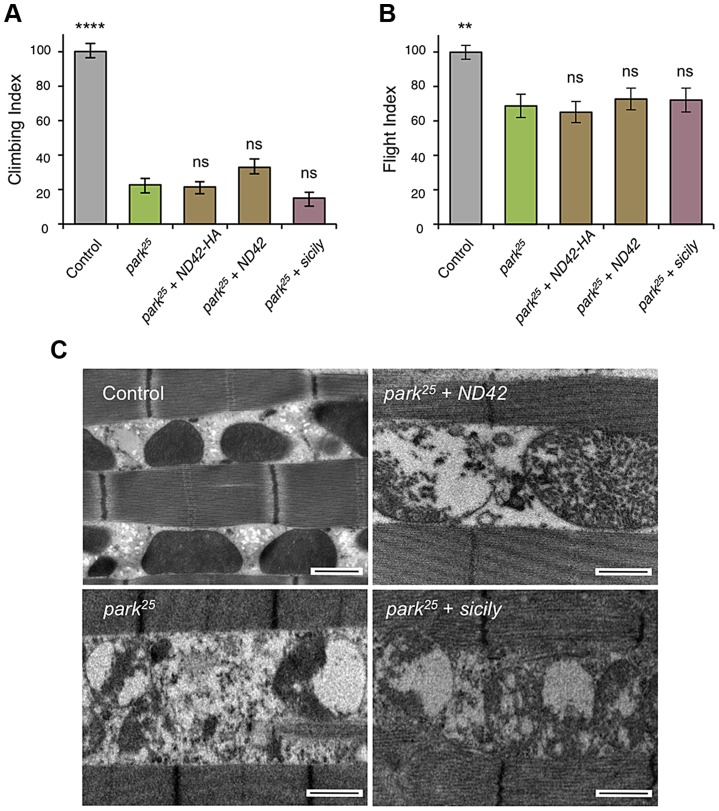
*ND42* or *sicily* overexpression does not rescue *parkin* mutant phenotypes in flies. In *park^25^* mutants, climbing (A) and flight ability (B), normalized to control, is not rescued by *ND42* or *sicily* overexpression. Histograms indicate mean ± s.e.m. (C) Transmission electron microscopy of flight muscle shows widespread disruption of mitochondrial integrity. Scale bar  = 1 µm. Overexpression was driven by the ubiquitous driver *da-GAL4*. Control genotype is *da-GAL4*/+. Number of animals tested, n>50. * *P*<0.05, *** *P*<0.001, **** *P*<0.0001, One-way ANOVA with Bonferroni correction. Comparisons are with *pink1^B9^* mutants.

Genetic interaction studies in *Drosophila* have linked *pink1* and *parkin* in a common pathway with *pink1* acting upstream of *parkin* [3], [6], [11]. To further characterize the putative action of ND42/NDUFA10 in the PINK1-Parkin pathway, we tested whether *ND42* overexpression could also rescue *parkin* mutants. Surprisingly, we found that overexpression of *ND42* was not able to rescue any *parkin* phenotypes tested, including locomotor behaviors, muscle and mitochondrial integrity, and male sterility (Figures 4A–4C, and S2B).

Recently it was reported that *Drosophila* sicily acts as a co-chaperone to bind and stabilize ND42 in the cytoplasm, promoting its mitochondrial import and the formation of CI [29]. Supporting a potential role for *sicily* in pink1 function, we found that knockdown of *sicily* in *Drosophila* cells phenocopied *pink1* mitochondrial hyperfusion (Figure S3). Since sicily promotes ND42 stability, and overexpression of *ND42* can rescue *pink1* mutant phenotypes, we hypothesized that *sicily* overexpression may also rescue *pink1* mutants. Indeed, we found that *sicily* overexpression rescued *pink1* mutant locomotor and mitochondrial phenotypes comparable to *ND42* overexpression (Figures 3A–3E). Also, overexpression of sicily failed to rescue similar *parkin* mutant phenotypes (Figures 4A–4C), mirroring the effects of ND42. Together these results demonstrate a genetic interaction of *ND42* and *sicily* with *pink1* but not *parkin*.

### NDUFA10 minimally affects Parkin translocation and mitophagy

**Figure 5:**
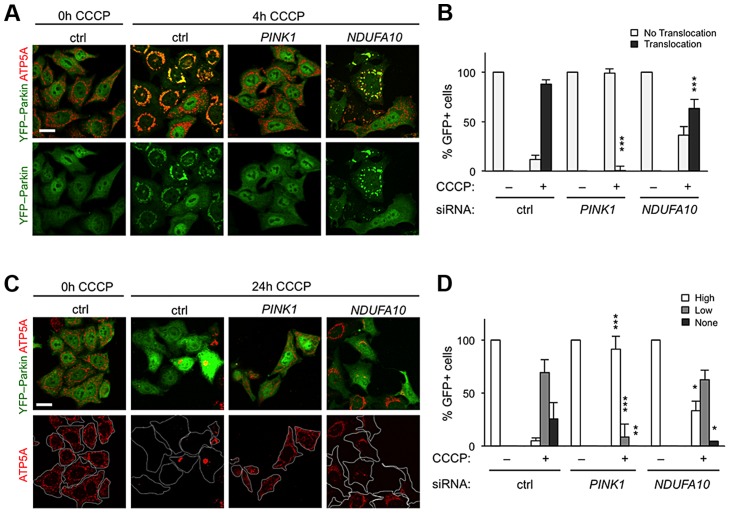
*NDUFA10* knockdown slightly reduces CCCP-induced Parkin translocation and mitophagy. (A) In HeLa cells stably transfected to express YFP-Parkin, before CCCP toxification (0 h) YFP-Parkin (green) has a diffuse cytoplasmic distribution in control (ctrl) siRNA treated cells. Following 4 h CCCP, YFP-Parkin co-localizes with mitochondria labeled with ATP5A immunostaining (red). *PINK1* siRNA treatment almost completely abolishes YFP-Parkin translocation. (B) Quantification of YFP-Parkin translocation as in A. (C) Stably transfected HeLa cells expressing YFP-Parkin, before CCCP treatment (0 h, ctrl) have a normal (“High”) mitochondrial content. Following 24 h treatment with CCCP, a high proportion of control cells (ctrl) show complete degradation (“none”) or perinuclear aggregated (“low”) mitochondria, visualized by ATP5A immunostaining (red). *PINK1* siRNA treatment almost completely abolishes mitophagy. (D) Quantification of mitochondrial content as in C. Histograms indicate mean ± s.d. of triplicate experiments. n>30 cells per experiment. Scale bar  = 20 µm. * *P<*0.05, ** *P<*0.01, *** *P<*0.001, Student's *t*-test compared with respective conditions in control siRNA.

The fact that *ND42* overexpression can rescue *pink1* but not *parkin* mutants would be consistent with it acting in a common pathway between PINK1 and Parkin. An intensively studied field of PINK1-Parkin function is the autophagic turnover of mitochondria, termed mitophagy [30]. In HeLa cells overexpressing YFP-Parkin, depolarization of the mitochondrial membrane with the protonophore carbonyl cyanide 3-chlorophenylhydrazone (CCCP) causes a rapid stabilization of PINK1 on the outer mitochondrial membrane, which stimulates the re-distribution of cytoplasmic Parkin to co-localize with mitochondria (Figures 5A and 5B). Prolonged exposure to CCCP induces substantial degradation of the mitochondria (Figures 5C, 5D, and S4). These phenomena are almost completely abolished by *PINK1* knockdown (Figures 5A–5D) [18]–[20], [22].

We next analyzed the effect of the human homolog of *ND42*, *NDUFA10*, on Parkin translocation and mitophagy. We found that *NDUFA10* knockdown had a modest but significant effect on Parkin translocation, though clearly not as much as loss of *PINK1* (Figures 5A, 5B, S1 and S5). Moreover, loss of *NDUFA10* only very minimally reduced the degree of mitophagy (Figures 5C, 5D, and S6). We also found no effect of *NDUFA10* knockdown on PINK1 stabilization following mitochondrial depolarization (Figure S7). These data indicate that *NDUFA10* does not play a significant role in PINK1/Parkin mediated mitophagy and suggests that the *in vivo* rescue was unlikely via the mitophagy pathway.

**Figure 6:**
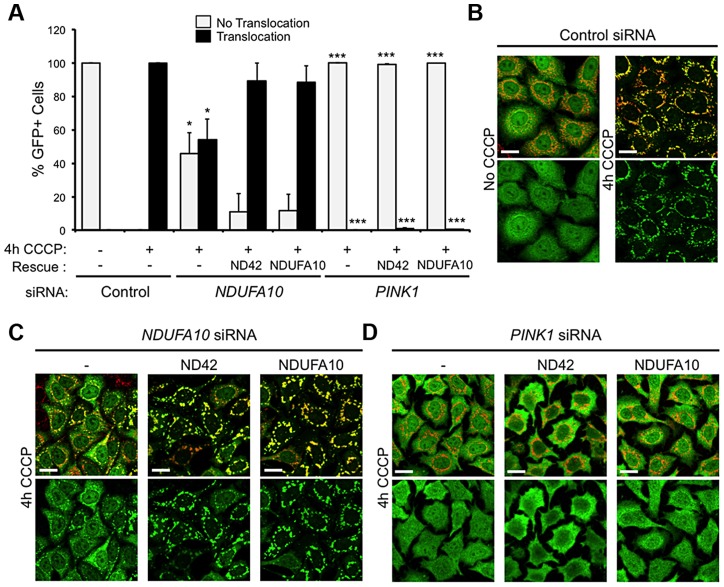
*NDUFA10/ND42* overexpression does not restore CCCP-induced Parkin translocation in the absence of *pink1*. (A) Quantification of the percentage of cells showing mitochondrially localized Parkin following 4 hours CCCP treatment of HeLa cells stably expressing YFP-Parkin transfected with (B) control siRNA, (C) *NDUFA10* siRNA, or (D) *PINK1* siRNA treatment and transfection with *NDUFA10* or fly *ND42* expression constructs. Scale bar  = 20 µm. * *P*<0.05, *** *P*<0.001, One-way ANOVA with Bonferroni correction compared to respective control siRNA treatment.

To further exclude a role for mitophagy in the rescue of *pink1* mutants, we assessed *in vitro* whether *NDUFA10* overexpression could promote CCCP-induced Parkin translocation in the absence of *PINK1*. Encouragingly, re-expression of either *NDUFA10* or *ND42* almost completely restored Parkin translocation reduced by *NDUFA10* knockdown (Figures 6A and 6C). However, when Parkin translocation was completely blocked by loss of *PINK1*, this was not rescued by expression of either *NDUFA10* or *ND42* (Figures 6A and 6D). These results support the idea that the rescue seen *in vivo* is unlikely due to activated mitophagy, raising the question of what mechanism is responsible.

### *ND42* overexpression restores CI activity in *pink1* mutants

**Figure 7:**
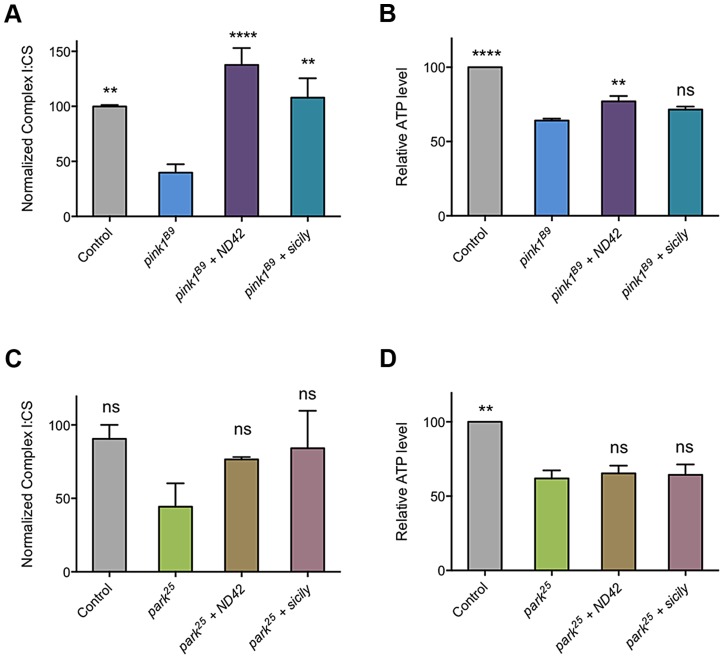
*ND42* and *sicily* overexpression can rescue complex I and ATP deficiencies in *pink1* but not *parkin* mutant flies. *ND42* or *sicily* was overexpressed in (A, B) *pink1^B9^* mutants or (C, D) *park^25^* mutants. Charts show (A, C) the ratio of complex I to citrate synthase (CS) activity, and (B, D) relative ATP levels, normalized to control. Histograms indicate mean ± s.e.m. Overexpression was driven by the ubiquitous driver *da-GAL4*. Control genotype is *da-GAL4*/+. ** *P*<0.01, **** *P*<0.0001, One-way ANOVA with Bonferroni correction. Comparisons are with *pink1^B9^* or *park^25^* mutants, as appropriate.

As loss of *PINK1* has been reported to cause decreased CI activity [24], we reasoned that suppression of *pink1* mutants by *ND42* overexpression may be due to restoration of CI activity. As previously reported, we observed a CI deficiency in *pink1* mutant flies, leading to decreased ATP production (Figures, 7A and 7B). We found that *ND42* overexpression was indeed able to completely restore both CI activity and ATP levels *in vivo* (Figure 7A and 7B). Extending these analyses to *parkin* mutants, we saw a non-significant decrease in CI activity in *parkin* mutants that remained unchanged by *ND42* overexpression (Figure 7C). Similarly, the significant depletion of ATP evident in *parkin* mutants was not rescued by *ND42* overexpression (Figure 7D), consistent with a lack of phenotypic rescue by *ND42* overexpression. Interestingly, we also found that *sicily* overexpression was able to completely restore CI activity in *pink1* mutants (Figure 7A), while the increase in ATP levels was not significant (Figure 7B). Similar to *ND42*, *sicily* overexpression had no effect in *parkin* mutants (Figures 7C and 7D).

### Analysis of ND42 Ser-250 phospho-variants in the rescue of *pink1* mutant locomotion and CI activity

While this work was in preparation, Morais et al [25] reported that NDUFA10 lacked phosphorylation at serine-250 in the absence of PINK1, and that expression of phospho-mimetic NDUFA10/ND42 specifically reversed *PINK1* deficits in various model systems, including restoring CI activity in mammalian systems and synaptic phenotypes in *Drosophila pink1* mutants. Our preceding data concur that overexpression of *ND42* can rescue some *pink1* mutant phenotypes (and not others), but interestingly we found that this can be achieved with expression of the wild type version without a specific requirement for the phospho-mimetic.

**Figure 8:**
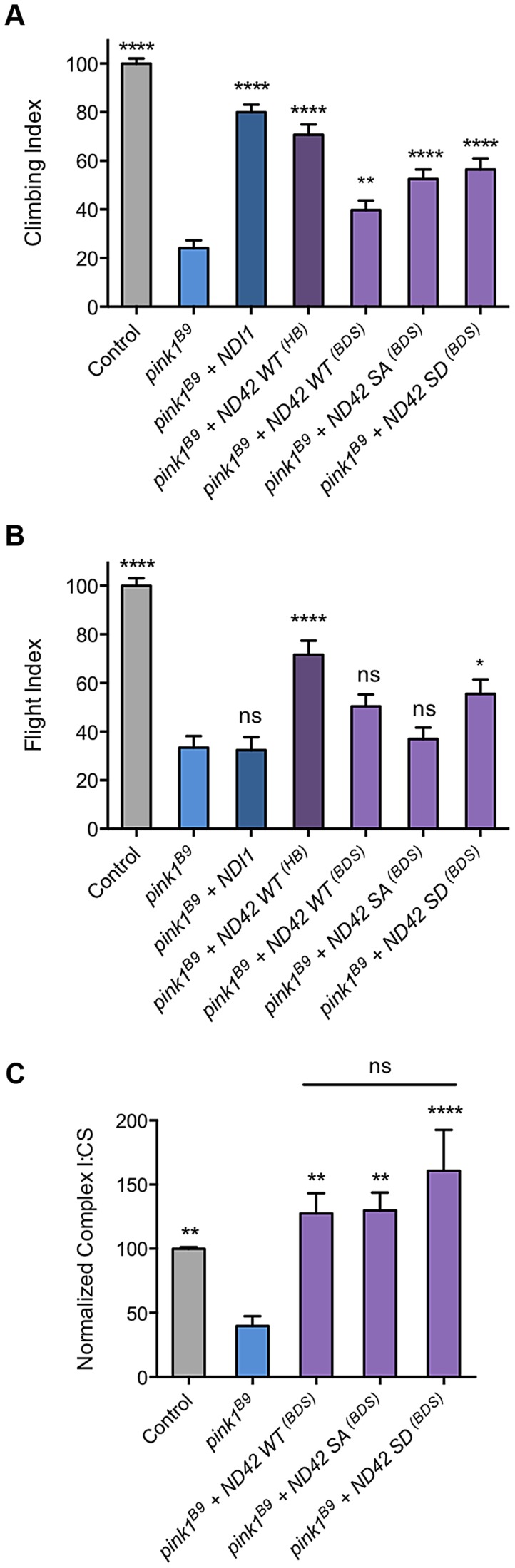
Analysis of ND42 Ser-250 phospho-variant rescue of *pink1* mutant climbing defect and complex I deficiency. Transgenes from different sources, labeled ‘HB’ [29] and ‘BDS’ [25], expressing wild type *ND42* (WT), non-phosphorylatable (SA) or phospho-mimetic (SD) variants of Ser-250 were tested for rescue of climbing (A), flight (B) and complex I (C) deficiencies in *pink1^B9^* mutants. For comparison, transgenic expression of the yeast complex I equivalent, *NDI1*, was also tested. Overexpression was driven by the ubiquitous driver *da-GAL4*. Control genotype is *da-GAL4*/+. * *P<*0.05, ** *P<*0.01, **** *P*<0.0001, One-way ANOVA with Bonferroni correction. Comparisons are with *pink1^B9^* mutants unless otherwise shown.

To further explore the potential role of Ser-250 phosphorylation in these assays, we tested in parallel our existing lines (previously reported in [31]) and those of Morais et al [25] and of the yeast equivalent of CI, *NDI1* [31]. As before, we found that expression of the previous WT transgene (designated *ND42^HB^*) significantly rescued *pink1* climbing and flight defects (Figures 8A and 8B). We also found that the Morais et al. transgenes (designated *ND42^BDS^*) expressing either WT or phospho-mimetic (SD) also partially rescued climbing, albeit to a lesser extent (Figure 8A). The WT version of these lines did not statistically improve flight ability whereas the SD did provide a modest rescue (Figure 8B). We also found that the non-phosphorylatable version (SA) provided significant rescue of climbing, equivalent to the phospho-mimetic, but again did not rescue flight (Figure 8A and 8B). Notably, in these assays *NDI1* expression significantly rescued climbing but not flight ability (Figure 8A and 8B), consistent with previous observations [31].

To better understand the relationship between the behavioral rescue and CI activity, and to assess functional efficacies of the various transgenic lines, we tested the ability of the phospho-variants transgenes to rescue the CI deficiency in *pink1* mutants. We found that expression of all phospho-variants were able to fully restore CI activity in *pink1* mutants (Fig. 8C). The degree of rescue was similar to that seen with the previous WT transgene (Fig. 7A), consistent with an equivalent level of expression between these lines (Fig. S1G). Interestingly, while we see the highest level of CI activity with the phospho-mimetic (SD), we also see a substantial rescue by the phopho-null (SA) in this *in vitro* assay.

### Overexpression of *parkin* rescues *pink1* mutants but does not restore CI activity

**Figure 9:**
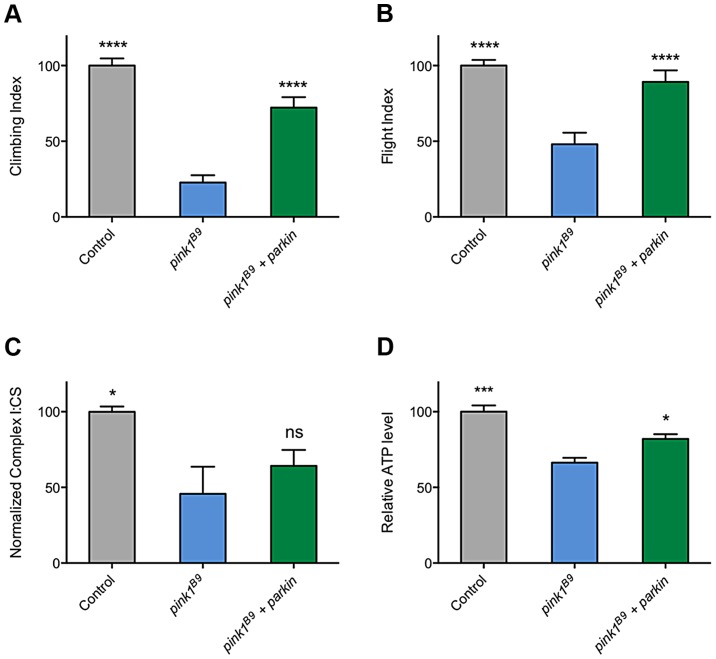
Overexpression of *parkin* can rescue behavioral phenotypes in *pink1* mutants but not complex I deficiency. Analysis of (A) climbing and (B) flight ability in *pink1^B9^* mutants overexpressing *parkin*. Charts show (C) the ratio of complex I to citrate synthase (CS) activity, and (D) relative ATP levels, normalized to control. Histograms indicate mean ± s.e.m. Overexpression was driven by the ubiquitous driver *da-GAL4*. Control genotype is *da-GAL4*/+. * *P<*0.05, ** *P<*0.01, *** *P<*0.001, **** *P*<0.0001, One-way ANOVA with Bonferroni correction. Comparisons are with *pink1^B9^* mutants.

The ability of multiple transgenes that restore CI activity to at least partially rescue climbing behavior supports the idea that promoting CI activity is differentially beneficial in *pink1* mutants but not *parkin* mutants and may hint at different underlying causes of pathogenicity. However, a puzzling aspect of this is the long-standing observations that *parkin* overexpression is sufficient to almost completely rescue many *pink1* mutant phenotypes (Figures 9A and 9B, and [3], [11], [32], [33]). Since, to our knowledge, it had never been reported, we tested whether the rescue may be due to restoration of CI activity in *pink1* mutants. Surprisingly, we found that *parkin* overexpression mildly improved ATP levels but did not restore CI function (Figures 9C and 9D). Hence, these genetic interactions further support independent and separable functions of PINK1 and Parkin.

## Discussion

### PINK1 and complex I function

PINK1 and Parkin have long been genetically linked in a common pathway that promotes mitochondrial homeostasis at least partly by directing the autophagic degradation of dysfunctional mitochondria as a mechanism of mitochondrial quality control. While this model potentially explains the occurrence of CI deficiency, oxidative stress, calcium dysregulation and elevated mtDNA mutations seen in patient tissues, and the age-related onset of PD [34], other models have been proposed to explain the pathological consequences of PINK1 and Parkin deficiency. Moreover, many mechanistic details by which the PINK1-Parkin pathway functions remain unexplained. To address these matters, we conducted an RNAi screen to identify genes whose loss-of-function either phenocopied or suppressed a *pink1* RNAi phenotype. We have identified a number of genes that fulfill these criteria (discussed below) but focused our current investigation on *ND42/NDUFA10* given the extensive literature implicating CI deficiency in PD pathogenesis and the fact that CI deficiency has previously been reported in *PINK1* mutant models and patient samples.

Loss of *ND42*/*NDUFA10* phenocopies the effect of *pink1* loss on mitochondrial morphology in *Drosophila* cells, and *ND42* overexpression rescues the *pink1* mutant phenotypes. However, *NDUFA10* knockdown has only modest effects on mitophagy, supporting a separate link between CI and PINK1 function. The simplest interpretation of these findings is that PINK1 normally regulates ND42/NDUFA10 abundance or activity through direct phosphorylation. Indeed, it was recently reported that NDUFA10 lacks phosphorylation at Ser-250 in *Pink1^-/-^* cells [25], although it remains to be determined whether PINK1 directly or indirectly regulates NDUFA10 phosphorylation. Moreover, it was reported that expression of a phospho-mimetic version of ND42/NDUFA10 specifically rescued phenotypes in multiple *PINK1* deficient systems, while an S250A mutant version of ND42/NDUFA10 that is incapable of being phosphorylated was unable to confer rescue. Consistent with this we find that, from equivalent expression levels, the phospho-mimetic (SD) provides a slightly better phenotypic rescue than the other variants, and likewise promotes a higher CI activity. Nevertheless, our results also show that the non-phosphorylatable S250A version is still able to restore CI activity and significantly rescue the climbing deficit in *pink1* mutant flies.

While further studies are needed to clarify the functional relationship between PINK1 and NDUFA10 in the regulation of CI, our findings provide further support mounting evidence that many manipulations that promote CI activity – overexpression of *NDUFA10*, *sicily*, *heix*, *Ret*, *dNK*, *TRAP1* and *NDI1*, or treatment with vitamin K, deoxynucleosides or folic acid [25], [31], [35]–[38] – can rescue *pink1* mutants, suggesting a more general defect underlies CI deficiency in loss of *pink1*. We hypothesize that the loss of CI activity in *pink1* mutants may be due to a general de-stabilization of CI. Assembly is a particular challenge for such a large, multi-subunit complex and occurs in a stepwise process that is highly regulated by many factors [39]. Even its association with other ETC complexes in supercomplexes affects CI's stability [40]. There is evidence for reduced complex stability in *pink1* mutants, though this may not be specific to CI [37], [41], [42]. One possibility is that PINK1 influences CI stability by directly promoting the assembly of CI, which may be regulated by NDUFA10.

The current findings also further support that the mechanism by which PINK1 influences CI activity appears to be separable from its well-characterized role in mitophagy, since, in agreement with some studies [24], [31] but in contrast to others [4], [8], [9], we do not find clear evidence of CI deficiency in *parkin* mutants flies. Moreover, it was unexpected to find that overexpression of *parkin* does not rescue the CI deficiency in *pink1* mutants, because substantial previous work has shown that parkin overexpression rescues all of the other *pink1* phenotypes, and because a prediction of the PINK1-parkin mitophagy pathway is that activation would trigger the selective removal of mitochondria deficient in CI activity. This finding suggests that CI deficiency alone cannot fully account for adult locomotor phenotypes seen in *pink1* mutants. Further studies are needed to clarify full spectrum of cellular defects in *pink1* and *parkin* mutants and their relative importance to the pathologic mechanism.

### Consideration of other screen hits

The present screen analyzed the effect of ∼600 genes comprising mostly genes with homology to kinase or phosphatase domains. Other hits from this screen, identifying both phenocopiers and suppressors, could also be attractive candidates as potential new factors of pink1/Parkin function. Notably several hits play a role in lipid biology. This is particularly noteworthy in light of our recent report that another RNAi screen identified the master regulator of lipogenesis, *SREBF1*, to affect pink1/Parkin-mediated mitophagy [43].

The phenocopier *Sphingosine kinase 1* and the suppressor *easily shocked* (encoding *Drosophila* Ethanolamine Kinase) are involved in phospholipid metabolic pathways. Sphingosine kinase catalyzes the production of sphingosine-1-phosphate (S1P), a key signaling molecule affecting cell growth and survival [44]. While S1P affects many cellular processes perhaps the most intriguing is its role in calcium mobilization from the endoplasmic reticulum (ER) [45] since Parkin was recently shown to promote ER-mitochondrial calcium transfer [23]. Interestingly, the breakdown of S1P generates phosphoethanolamine, the enzymatic product of ethanolamine kinase and a precursor metabolite of the key phospholipid phosphatidylethanolamine (PE). Loss of mitochondrial PE has been shown to affect mitochondrial morphology, oxidative phosphorylation and even the formation of complex I-containing supercomplexes [46]. The identification of *four wheel drive* (encoding *Drosophila* phosphatidylinositol 4-kinase beta), which catalyzes the formation of PI(4)P, is also intriguing since mutations in *SYNJ1*, which encodes PI(4,5)P_2_ phosphatase, were identified in families with early onset parkinsonism [47], [48].

Also related to lipid biology is Nocturnin although a direct link to mitochondria biology is less obvious. Nocturnin encodes a circadian deadenylase thought to be involved in the rhythmic regulation of gene expression by removal of polyA tails from mRNAs. Mice lacking Nocturnin are resistant to diet-induced obesity and hepatic steatosis [49], linking its function to lipid metabolism. Further studies will be needed to determine the extent to which lipids in general or specific lipids, and their regulated synthesis, impact on pink1/Parkin biology and regulation of mitochondrial dynamics and quality control. Nevertheless, this screen provides a resource for characterizing novel factors that regulate mitochondrial morphology.

## Materials and Methods

### Cell culture

*Drosophila* S2R+ cells were cultured in Schneider's medium (Gibco) containing 10% (vol/vol) heat-inactivated fetal bovine serum (Sigma), Penicillin 10 units/ml (Sigma) and Streptomycin 10 µg/ml (Sigma). Cells were maintained in a 25°C incubator. HeLa cells were cultured in DMEM GLUTAMAX media (Gibco) containing 10% (vol/vol) heat-inactivated fetal bovine serum (Sigma), Penicillin 10 units/ml (Sigma) and Streptomycin 10 µg/ml (Sigma). Cells were maintained in a 37°C incubator with 5% CO_2_. A stable transfected HeLa cell line expressing YFP-Parkin in pLVX-puro was cultured in DMEM GLUTAMAX media (Gibco) containing 10% (vol/vol) heat-inactivated fetal bovine serum (Sigma), Penicillin 10 units/ml (Sigma) and Streptomycin 10 µg/ml (Sigma). Cells were maintained in a 37°C incubator with 5% CO_2_.

### RNAi screening and high-content microscopy

The kinome/phosphatome sub-library was generated based upon the second generation *Drosophila* dsRNA library (Heidelberg 2). Detailed information on amplicon targets is available online (http://rnai-screening-wiki.dkfz.de/signaling/wiki/display/rnaiwiki/Drosophila+RNAi+libraries). This sub-library was designed to contain all known and computed kinases and phosphatases, genes with some homology to these enzyme classes, but also some other genes of general interest. Library dsRNAs were plated at 250 ng in 5 µl of H_2_O into Perkin Elmer 384 well view plate (Product number: 6007470). Screen plates were arrayed with the inclusion of gaps to allow for the addition of user-specific controls. Here we added dsRNAs targeting *Marf*, *Drp1*, *OPA1*, *Fis1*, *pink1* and *parkin*. These controls consistently all showed the expected results. For the *pink1* RNAi background, 250 ng dsRNA against *pink1* was added to each well prior to screening. A ‘double dose’ of dsRNA did not appear to affect mitochondrial morphology (see below). 15,000 *Drosophila* S2R+ cells were added to each well in 30 µl of Schneider's medium (Gibco) without FBS (Sigma). Plates were incubated at 25°C for 1 hour in which time dsRNAs are taken up by the cells. Following this incubation 30 µl of Schneider's medium (Gibco) containing 20% FBS (v/v) was added. The plates were then sealed and incubated at 25°C incubator for 4 days. Cells were stained with 100 nM Mitotracker Red (Invitrogen, M7512) and 20 µg/ml Hoechst 33342 (Invitrogen, H3570) for 15 minutes. Media was replaced and imaging was performed on live cells. Imaging was performed on an IN Cell Analyzer 1000 (GE Healthcare) automated microscope using a 40× air objective (Nikon, 0.60 NA).

### Mitochondrial morphology analysis

Cells were prepared identically as for the high-throughput screening conditions, except for wild type where background 250 ng dsRNA targeting *DsRed* was added to each condition to mirror the ‘double dose’ of dsRNA in the *pink1* background. Cells were imaged live under ambient conditions on a Deltavision RT deconvolution wide field microscope (Olympus, 100× objective, 1.4 NA) using 8 well µ-Slides (Ibidi), with 10 images taken per condition. Cells were scored for their gross mitochondrial morphology by eye with the scorer blinded to the experimental conditions. Where rotenone (20 µM) was used, cells were treated for 2 hours before mitochondrial morphology was analysed. A score would be assigned for a whole field of view and an average score would be calculated for the 10 images per condition. A score of 1 would be given to a field of view that had mainly fragmented mitochondria. A score of 2 would be given to a field of view that had a mainly wild type mitochondrial network with a mix of short-round and long-tubular mitochondria. A score of 3 would be given to a field of view that had mainly tubular mitochondria. A score of 4 would be given to a field of view that had mainly clumped mitochondria where the mitochondria had formed a single or few large peri-nuclear clusters.

### RNAi reagents

*Drosophila* gene dsRNAs were generated using the MEGAscript T7 Kit (Ambion), using T7-flanked DNA amplicons from the library as template. Control dsRNA for *Drosophila* cell qRT-PCR analysis was a 782 bp sequence targeted against *C. elegans* gene *R06F6.2* which has no ∼21mer homology within the *Drosophila* genome. siRNAs targeting human genes were obtained from the siGENOME SMARTpool collection (Dharmacon) as follows: control siRNA is Non-Targeting siRNA Pool #1 (product code; D-001206-13-20); *PINK1* (product code; M-004030-02); *NDUFA10* (product code; M-006752-00).

### Quantitative real-time PCR

Total RNA from live cells was prepared from three replicates of each dsRNA treatment using RNeasy (Qiagen). RNA concentration was then determined spectrophotometrically. Once treated with DNase, total RNA was reverse-transcribed using RETROscript (Ambion) or Protoscript (NEB) according to the manufacturer's protocol. Quantitative PCR was performed using SYBR Green (Sigma) on a MyIQ real time PCR detection system (Bio-Rad). Each PCR included three technical replicates, which were repeated as three biological replicates. Levels for each transcript were normalized to a 18S rRNA (*Drosophila*: *18SrRNA*; Human: *RNA18S5*) control by the 2^-ΔΔ*CT*^ method.

For *Drosophila* genes, primers used were:

18S - Forward: TCTAGCAATATGAGATTGAGCAATAAG

18S - Reverse: AATACACGTTGATACTTTCATTGTAGC

pink1 - Forward: GACGACCCTCGCACATAA

pink1 - Reverse: AACAGTCCGGAGATCCTACAG

ND42 - Forward: CGTTTCGATGTCCCGGAGCT

ND42 - Reverse: GTCTGCATTGTAGCCAGGAC

CG7712 - Forward: CGCAATGTGACCGACATCCG

CG7712 - Reverse: CGCATGATATGGCCTTCTG

For human genes, primers used were:

18S - Forward: CAGCCACCCGAGATTGAGCA

18S - Reverse: TAGTAGCGACGGGCGGTGTG

PINK1 - Forward: GCCGGACGCTGTTCCTCGTT

PINK1 - Reverse: TGGACACCTCTGGGGCCATC

NDUFA10 - Forward: GATCCGAGAAGCAATGATG

NDUFA10 - Reverse: TGGAGCGCTCCAACACAACA

### Antibodies

The following primary antibodies were used, mouse anti-ATP5A (MS507, MitoSciences; 1∶2000), rabbit anti-GFP (ab6556, Abcam; 1∶5000). Secondary antibodies used were rabbit polyclonal anti-mouse IgG-H&L (DyLight 594, Invitrogen; 1∶5000) and goat anti-rabbit IgG (Alexa Fluor 488, Invitrogen; 1∶5000).

### Parkin translocation and mitophagy assays

YFP-Parkin HeLa cells were reverse-transfected with siRNAs using DharmaFECT 1 (Dharmacon). For Parkin translocation, cells were incubated for 4 days then treated with 10 µM CCCP or equivalent volume of the solvent (EtOH) for 4 hours. For mitophagy, cells were incubated for 3 days then treated with 10 µM CCCP or equivalent volume of the solvent (EtOH) for 24 hours. Cells were fixed in ice-cold methanol for 10 minutes and washed in PBS. Mitochondrial staining was achieved by using anti-ATP5A antibody. Imaging was performed on an Olympus FV1000 confocal microscope (Olympus, 60× oil objective, 1.25 NA). For Parkin translocation, cells were scored for the accumulation of YFP-Parkin on mitochondria. For mitophagy, cells were scored for mitochondrial load based on having a normal load of mitochondria, few mitochondria or no mitochondria. At least 20 cells were scored per treatment and 3 biological replicates were performed.

### PINK1 accumulation assay

HeLa cells were reverse-transfected with siRNAs using DharmaFECT 1. After 3 days cells were transfected with PINK1-EGFP using Effectene (Qiagen). After a further 1 day cells were treated with 10 µM CCCP or equivalent volume of the solvent (EtOH) for 1 hour before fixation with ice-cold methanol for 10 minutes and washed in PBS. Immunofluorescence was performed using anti-ATP5A and anti-GFP antibodies, and appropriate fluorescent secondary antibodies. Imaging was performed on an Olympus FV1000 confocal microscope. Cells were scored for the accumulation of PINK1-EGFP on mitochondria. At least 20 cells were scored per treatment and 3 biological replicates were performed.

### TMRM assay

TMRM assay to measure mitochondrial membrane polarity was done as previously described [8]. Briefly, HeLa cells were reverse-transfected with siRNAs using DharmaFECT for 4 days. Cells were then treated with 10 µM CCCP or equivalent volume of the solvent (EtOH) for 1 hour. Cells were incubated with 50 nM TMRM (VTX668, Fisher) in PBS with 10 µM CCCP or equivalent volume of solvent for 30 minutes, then washed in PBS with 10 µM CCCP or equivalent volume of solvent 5 times. TMRM fluorescence was read on a spectrophotometer at 550 nm (Berthold technologies Mithras LB940). Triplicate readings were taken from 3 biological replicates.

### *Drosophila* genetics

*Drosophila* were raised under standard conditions at 25°C on food consisting of agar, cornmeal and yeast. *pink1^B9^* mutants [11] were provided by J. Chung (KAIST). *park^25^* mutants, fertility tests, flight and climbing assays were performed as previously described [6], [50]. *w^1118^* and *da-GAL4* strains were obtained from the Bloomington *Drosophila* Stock Center (Bloomington, IN). UAS-*ND42*-RNAi lines (GD: 14444; KK: 101787) were obtained form the Vienna *Drosophila* Resource Centre [51]. UAS-*ND42*, UAS-*ND42*-HA and UAS-*sicily* have been described previously [29] and were a gift from H. Bellen (Baylor College of Medicine). The additional UAS-*ND42* lines (WT, SA and SD) from Morais et al. [25] were provided by Patrik Verstreken.

### Measurement of complex I activity

Mitochondria-enriched fractions were prepared from whole adult male flies (∼3 days old) with the indicated genotype (10 flies were used for each sample). Flies were gently crushed in chilled isolation buffer (250 mM sucrose, 10 mM Tris-HCl, pH 7.4, 0.15 mM MgCl_2_) using a plastic pestle homogenizer, then centrifuged twice at 500×g for 5 minutes at 4°C to remove debris. The supernatant was centrifuged 5000×g for 5 minutes at 4°C. The resulting pellet containing mitochondria was re-suspended in the isolation buffer or assay buffers. Complex I activity was measured using a modified method from Birch-Machin et al [52]. Briefly, samples were subjected to 3 cycles of freeze-thaw in liquid nitrogen. Complex I activity was determined by following the oxidation of NADH at 340 nm with a reference wavelength of 425 nm (ε = 6.22 mM^−1^ cm^−1^) at 30°C using a BMG Labtech FLUOStar plate reader. The assay buffer contained 25 mM KH_2_PO_4_, 5 mM MgCl_2_, (pH 7.2), 3 mM KCN, 2.5 mg per ml BSA, 50 µM ubiquinone, 2 µg/ml antimycin A and mitochondrial extract. The baseline was recorded for 5 minutes and the reaction was started with 125 µM NADH measured for 30 minutes, 15 µg/ml rotenone was added to inhibit the reaction and measured for 15 minutes. The results are expressed as µmol NADH oxidised/min/citrate synthase activity. Citrate synthase was measured by following the production of 5-thio-2-nitrobenzoate at 30°C using a BMG Labtech FLUOStar plate reader after samples had undergone 3 cycles of freeze-thaw in liquid nitrogen. The assay buffer was 100 mM Tris HCl (pH 8.0), 0.1 mM DTNB, 50 µM acetyl Coenzyme A, 0.1% Triton X-100 and mitochondrial extract per well. The baseline was recorded for 5 minutes at 412 nm, then the reaction was started by the addition of 0.5 mM oxaloacetate acid and the rate was recorded for 15 minutes.

### ATP assays

Five male flies (3 days old) were homogenized in 100 µl of 6 M guanidine-HCl in extraction buffer (100 mM Tris, 4 mM EDTA, pH 7.8) to inhibit ATPases. Homogenized samples were subjected to rapid freezing in liquid nitrogen, followed by boiling for 3 min. Samples were cleared by centrifugation, and supernatant was diluted (1/100) with extraction buffer and mixed with a luminescent solution (CellTiter-Glo Luminescent Cell Viability Assay, Promega, Fitchburg, WI, USA). Luminescence was measured on a Varioskan™ Flash Multimode Reader (Thermo Scientific, Waltham, MA, USA). The relative ATP levels were calculated by dividing the luminescence by the total protein concentration, which was determined by the Bradford method.

### Statistical analyses

For Parkin translocation, mitophagy and PINK1 stabilization, statistical significance was calculated by using Student's *t*-test on triplicate experiments comparing against control siRNA/dsRNAs of the equivalent experimental condition. Biochemical and behavioral assays in *Drosophila* were analyzed by one-way ANOVA with Bonferroni correction. Male fertility was analyzed by Chi-square test.

## Supporting Information

Figure S1 Quantitative real-time PCR measurement for relative gene expression in RNAi knockdown and transgenic overexpression conditions. (A–C) *Drosophila* S2R+ cells treated with dsRNAs for the indicated genes. Message abundance of the respective gene is shown relative to control dsRNA treatment. (D–E) HeLa cells transfected with the indicated siRNAs. Message abundance of the respective gene is shown relative to control (ctrl) siRNA treatment. (F) The four *NDUFA10* SMARTpool siRNAs were tested individually and compared to combined SMARTpool and shown relative to ctrl. Transcript levels are normalized against a housekeeping gene, *18S rRNA*. All differences are highly significant (*P*<0.001) compared to the relevant control. (G, H) Relative expression levels in flies overexpressing *ND42* or *sicily*, as indicated, driven by *da-GAL4*. ** *P<*0.01, *** *P<*0.001, one-way ANOVA with Bonferroni correction (G) or Student's *t*-test (H) compared with control genotype (*da-GAL4*/+).
(TIFF)

Figure S2 *ND42* or *sicily* overexpression does not rescue *pink1* or *parkin* mutant male sterility. *ND42* or *sicily* was overexpressed by *da-GAL4* in either *pink1* (A) or *parkin* (B) mutant males and fertility was assessed (n>45 males of each genotype). Fertility was almost completely restored by *da-GAL4* induced re-expression of *pink1* or *parkin*. Control genotype is *da-GAL4*/+. **** *P*<0.0001, Chi-square test. Comparisons are with control genotype unless otherwise indicated.
(TIFF)

Figure S3 RNAi knockdown of *sicily* causes mitochondrial hyperfusion. (A) *Drosophila* S2R+ cells treated with indicated dsRNAs and stained with MitoTracker Red to visualize mitochondria. (B) Cells were scored as in Fig. 2 for relative mitochondrial morphology. Scale bar  = 5 µm. ** *P<*0.01, *** *P<*0.001, Student's *t*-test compared with control dsRNA.
(TIFF)

Figure S4 Categorization of mitochondrial content during mitophagy. HeLa cells transiently transfected to express YFP-Parkin (green) induce mitophagy following prolonged exposure to CCCP. Mitochondrial content can be monitored by ATP5A immunostaining (red). Cells with normal mitochondrial content, as seen before toxification, are categorized as “High”. Depolarized mitochondria become aggregated and in a perinuclear region, and termed “Low”. Cells which have undergone complete mitophagy have a mitochondrial content scored as “None”.
(TIFF)

Figure S5 Individual *NDUFA10* siRNAs attenuate CCCP-induced Parkin translocation. (A) In HeLa cells stably transfected to express YFP-Parkin, before CCCP toxification (0 h) YFP-Parkin (green) has a diffuse cytoplasmic distribution in control (ctrl) siRNA treated cells. Following 4 h CCCP, YFP-Parkin co-localizes with mitochondria labeled with ATP5A immunostaining (red). *PINK1* siRNA treatment almost completely abolishes YFP-Parkin translocation. Individual *NDUFA10* siRNAs, #1 and #3, significantly reduce YFP-Parkin translocation. (B) Quantification of YFP-Parkin translocation as in A, scored in triplicate experiments. n>30 cells per experiment. Scale bar  = 20 µm. *** *P<*0.001, Student's *t*-test compared with control siRNA.
(TIFF)

Figure S6 Individual *NDUFA10* siRNAs reduce CCCP-induced mitophagy. (A) Stably transfected HeLa cells expressing YFP-Parkin, before CCCP treatment (0 h, ctrl) have a normal (“High”) mitochondrial content. Following 24 h treatment with CCCP, a high proportion of control cells (ctrl) show complete degradation (“none”) or perinuclear aggregated (“low”) mitochondria, visualized by ATP5A immunostaining (red). *PINK1* siRNA treatment almost completely abolishes mitophagy. Individual *NDUFA10* siRNAs, #1 and #3, significantly reduce mitophagy. (B) Quantification of mitochondrial content as in A, scored in triplicate experiments. n>30 cells per experiment. Scale bar  = 20 µm. * *P<*0.05, ** *P<*0.01, *** *P<*0.001, Student's *t*-test compared with control siRNA.
(TIFF)

Figure S7 *NDUFA10* knockdown does not affect CCCP-induced pink1 stabilization. (A) HeLa cells transiently expressing PINK1-GFP and treated with control (ctrl) siRNA. Before CCCP toxification (0 h) PINK1-GFP (green) has a diffuse distribution. Following 1 h CCCP PINK1-GFP becomes stabilized and accumulates on mitochondria, labeled with ATP5A immunostaining (red). Boxed areas are shown magnified in images below. (B) Quantification of PINK1-GFP stabilization as in A. Charts indicate mean ± s.d. of triplicate experiments. n>15 cells per experiment. Scale bars; low mag.  = 20 µm, zoom  = 4 µm.
(TIFF)

## References

[1] Kitada T, Asakawa S, Hattori N, Matsumine H, Yamamura Y, et al (1998) Mutations in the parkin gene cause autosomal recessive juvenile parkinsonism. Nature 392: 605–608. 9560156 10.1038/33416

[2] Valente EM, Abou-Sleiman PM, Caputo V, Muqit MM, Harvey K, et al (2004) Hereditary early-onset Parkinson's disease caused by mutations in PINK1. Science 304: 1158–1160. 15087508 10.1126/science.1096284

[3] Clark IE, Dodson MW, Jiang C, Cao JH, Huh JR, et al (2006) Drosophila pink1 is required for mitochondrial function and interacts genetically with parkin. Nature 441: 1162–1166. 16672981 10.1038/nature04779

[4] Flinn L, Mortiboys H, Volkmann K, Koster RW, Ingham PW, et al (2009) Complex I deficiency and dopaminergic neuronal cell loss in parkin-deficient zebrafish (Danio rerio). Brain 132: 1613–1623. 19439422 10.1093/brain/awp108

[5] Gautier CA, Kitada T, Shen J (2008) Loss of PINK1 causes mitochondrial functional defects and increased sensitivity to oxidative stress. Proc Natl Acad Sci U S A 105: 11364–11369. 18687901 10.1073/pnas.0802076105 PMC2516271

[6] Greene JC, Whitworth AJ, Kuo I, Andrews LA, Feany MB, et al (2003) Mitochondrial pathology and apoptotic muscle degeneration in Drosophila parkin mutants. Proc Natl Acad Sci U S A 100: 4078–4083. 12642658 10.1073/pnas.0737556100 PMC153051

[7] Grunewald A, Gegg ME, Taanman JW, King RH, Kock N, et al (2009) Differential effects of PINK1 nonsense and missense mutations on mitochondrial function and morphology. Exp Neurol 219: 266–273. 19500570 10.1016/j.expneurol.2009.05.027

[8] Mortiboys H, Thomas KJ, Koopman WJ, Klaffke S, Abou-Sleiman P, et al (2008) Mitochondrial function and morphology are impaired in parkin-mutant fibroblasts. Ann Neurol 64: 555–565. 19067348 10.1002/ana.21492 PMC2613566

[9] Muftuoglu M, Elibol B, Dalmizrak O, Ercan A, Kulaksiz G, et al (2004) Mitochondrial complex I and IV activities in leukocytes from patients with parkin mutations. Mov Disord 19: 544–548. 15133818 10.1002/mds.10695

[10] Palacino JJ, Sagi D, Goldberg MS, Krauss S, Motz C, et al (2004) Mitochondrial dysfunction and oxidative damage in parkin-deficient mice. J Biol Chem 279: 18614–18622. 14985362 10.1074/jbc.M401135200

[11] Park J, Lee SB, Lee S, Kim Y, Song S, et al (2006) Mitochondrial dysfunction in Drosophila PINK1 mutants is complemented by parkin. Nature 441: 1157–1161. 16672980 10.1038/nature04788

[12] Ved R, Saha S, Westlund B, Perier C, Burnam L, et al (2005) Similar patterns of mitochondrial vulnerability and rescue induced by genetic modification of alpha-synuclein, parkin, and DJ-1 in Caenorhabditis elegans. J Biol Chem 280: 42655–42668. 16239214 10.1074/jbc.M505910200 PMC3910375

[13] Wood-Kaczmar A, Gandhi S, Yao Z, Abramov AY, Miljan EA, et al (2008) PINK1 is necessary for long term survival and mitochondrial function in human dopaminergic neurons. PLoS One 3: e2455. 18560593 10.1371/journal.pone.0002455 PMC2413012

[14] Poole AC, Thomas RE, Andrews LA, McBride HM, Whitworth AJ, et al (2008) The PINK1/Parkin pathway regulates mitochondrial morphology. Proc Natl Acad Sci U S A 105: 1638–1643. 18230723 10.1073/pnas.0709336105 PMC2234197

[15] Wang X, Winter D, Ashrafi G, Schlehe J, Wong YL, et al (2011) PINK1 and Parkin target Miro for phosphorylation and degradation to arrest mitochondrial motility. Cell 147: 893–906. 22078885 10.1016/j.cell.2011.10.018 PMC3261796

[16] Yang Y, Ouyang Y, Yang L, Beal MF, McQuibban A, et al (2008) Pink1 regulates mitochondrial dynamics through interaction with the fission/fusion machinery. Proc Natl Acad Sci USA 105: 7070–7075. 18443288 10.1073/pnas.0711845105 PMC2383971

[17] Ziviani E, Tao RN, Whitworth AJ (2010) Drosophila parkin requires PINK1 for mitochondrial translocation and ubiquitinates mitofusin. Proc Natl Acad Sci U S A 107: 5018–5023. 20194754 10.1073/pnas.0913485107 PMC2841909

[18] Matsuda N, Sato S, Shiba K, Okatsu K, Saisho K, et al (2010) PINK1 stabilized by mitochondrial depolarization recruits Parkin to damaged mitochondria and activates latent Parkin for mitophagy. J Cell Biol 189: 211–221. 20404107 10.1083/jcb.200910140 PMC2856912

[19] Narendra DP, Jin SM, Tanaka A, Suen DF, Gautier CA, et al (2010) PINK1 is selectively stabilized on impaired mitochondria to activate Parkin. PLoS Biol 8: e1000298. 20126261 10.1371/journal.pbio.1000298 PMC2811155

[20] Geisler S, Holmstrom KM, Skujat D, Fiesel FC, Rothfuss OC, et al (2010) PINK1/Parkin-mediated mitophagy is dependent on VDAC1 and p62/SQSTM1. Nat Cell Biol 12: 119–131. 20098416 10.1038/ncb2012

[21] Narendra D, Tanaka A, Suen DF, Youle RJ (2008) Parkin is recruited selectively to impaired mitochondria and promotes their autophagy. J Cell Biol 183: 795–803. 19029340 10.1083/jcb.200809125 PMC2592826

[22] Vincow ES, Merrihew G, Thomas RE, Shulman NJ, Beyer RP, et al (2013) The PINK1-Parkin pathway promotes both mitophagy and selective respiratory chain turnover in vivo. Proc Natl Acad Sci U S A 110: 6400–6405. 23509287 10.1073/pnas.1221132110 PMC3631677

[23] Cali T, Ottolini D, Negro A, Brini M (2013) Enhanced parkin levels favor ER-mitochondria crosstalk and guarantee Ca(2+) transfer to sustain cell bioenergetics. Biochim Biophys Acta 1832: 495–508. 23313576 10.1016/j.bbadis.2013.01.004

[24] Morais V, Verstreken P, Roethig A, Smet J, Snellinx A, et al (2009) Parkinson's disease mutations in PINK1 results in decreased Complex I activity and deficient synaptic function. EMBO Molecular Medicine 1: 99–111. 20049710 10.1002/emmm.200900006 PMC3378121

[25] Morais VA, Haddad D, Craessaerts K, De Bock PJ, Swerts J, et al (2014) PINK1 loss-of-function mutations affect mitochondrial complex I activity via NdufA10 ubiquinone uncoupling. Science 344: 203–207. 24652937 10.1126/science.1249161

[26] Poole AC, Thomas RE, Yu S, Vincow ES, Pallanck L (2010) The mitochondrial fusion-promoting factor mitofusin is a substrate of the PINK1/parkin pathway. PLoS One 5: e10054. 20383334 10.1371/journal.pone.0010054 PMC2850930

[27] Janssen RJ, Nijtmans LG, van den Heuvel LP, Smeitink JA (2006) Mitochondrial complex I: structure, function and pathology. J Inherit Metab Dis 29: 499–515. 16838076 10.1007/s10545-006-0362-4

[28] Vinothkumar KR, Zhu J, Hirst J (2014) Architecture of mammalian respiratory complex I. Nature. E-pub ahead of print doi:10.1038/nature13686 10.1038/nature13686 PMC4224586 25209663

[29] Zhang K, Li Z, Jaiswal M, Bayat V, Xiong B, et al (2013) The C8ORF38 homologue Sicily is a cytosolic chaperone for a mitochondrial complex I subunit. J Cell Biol 200: 807–820. 23509070 10.1083/jcb.201208033 PMC3601355

[30] Youle RJ, Narendra DP (2011) Mechanisms of mitophagy. Nat Rev Mol Cell Biol 12: 9–14. 21179058 10.1038/nrm3028 PMC4780047

[31] Vilain S, Esposito G, Haddad D, Schaap O, Dobreva MP, et al (2012) The yeast complex I equivalent NADH dehydrogenase rescues pink1 mutants. PLoS Genet 8: e1002456. 22242018 10.1371/journal.pgen.1002456 PMC3252300

[32] Exner N, Treske B, Paquet D, Holmstrom K, Schiesling C, et al (2007) Loss-of-function of human PINK1 results in mitochondrial pathology and can be rescued by parkin. J Neurosci 27: 12413–12418. 17989306 10.1523/JNEUROSCI.0719-07.2007 PMC6673250

[33] Yang Y, Gehrke S, Imai Y, Huang Z, Ouyang Y, et al (2006) Mitochondrial pathology and muscle and dopaminergic neuron degeneration caused by inactivation of Drosophila Pink1 is rescued by Parkin. Proc Natl Acad Sci USA 103: 10793–10798. 16818890 10.1073/pnas.0602493103 PMC1502310

[34] Exner N, Lutz AK, Haass C, Winklhofer KF (2012) Mitochondrial dysfunction in Parkinson's disease: molecular mechanisms and pathophysiological consequences. EMBO J 31: 3038–3062. 22735187 10.1038/emboj.2012.170 PMC3400019

[35] Klein P, Muller-Rischart AK, Motori E, Schonbauer C, Schnorrer F, et al (2014) Ret rescues mitochondrial morphology and muscle degeneration of Drosophila Pink1 mutants. EMBO J 33: 341–355. 24473149 10.1002/embj.201284290 PMC3983680

[36] Vos M, Esposito G, Edirisinghe JN, Vilain S, Haddad DM, et al (2012) Vitamin K2 is a mitochondrial electron carrier that rescues pink1 deficiency. Science 336: 1306–1310. 22582012 10.1126/science.1218632

[37] Tufi R, Gandhi S, de Castro IP, Lehmann S, Angelova PR, et al (2014) Enhancing nucleotide metabolism protects against mitochondrial dysfunction and neurodegeneration in a PINK1 model of Parkinson's disease. Nat Cell Biol 16: 157–166. 24441527 10.1038/ncb2901 PMC4199097

[38] Zhang L, Karsten P, Hamm S, Pogson JH, Muller-Rischart AK, et al (2013) TRAP1 rescues PINK1 loss-of-function phenotypes. Hum Mol Genet 22: 2829–2841. 23525905 10.1093/hmg/ddt132 PMC3690968

[39] Lazarou M, Thorburn DR, Ryan MT, McKenzie M (2009) Assembly of mitochondrial complex I and defects in disease. Biochim Biophys Acta 1793: 78–88. 18501715 10.1016/j.bbamcr.2008.04.015

[40] Acin-Perez R, Bayona-Bafaluy MP, Fernandez-Silva P, Moreno-Loshuertos R, Perez-Martos A, et al (2004) Respiratory complex III is required to maintain complex I in mammalian mitochondria. Mol Cell 13: 805–815. 15053874 10.1016/s1097-2765(04)00124-8 PMC3164363

[41] Liu W, Acin-Perez R, Geghman KD, Manfredi G, Lu B, et al (2011) Pink1 regulates the oxidative phosphorylation machinery via mitochondrial fission. Proc Natl Acad Sci U S A 108: 12920–12924. 21768365 10.1073/pnas.1107332108 PMC3150934

[42] Amo T, Saiki S, Sawayama T, Sato S, Hattori N (2014) Detailed analysis of mitochondrial respiratory chain defects caused by loss of PINK1. Neurosci Lett 580: 37–40. 25092611 10.1016/j.neulet.2014.07.045

[43] Ivatt RM, Sanchez-Martinez A, Godena VK, Brown S, Ziviani E, et al (2014) Genome-wide RNAi screen identifies the Parkinson disease GWAS risk locus SREBF1 as a regulator of mitophagy. Proc Natl Acad Sci U S A 111: 8494–8499. 24912190 10.1073/pnas.1321207111 PMC4060696

[44] Olivera A, Spiegel S (2001) Sphingosine kinase: a mediator of vital cellular functions. Prostaglandins Other Lipid Mediat 64: 123–134. 11324702 10.1016/s0090-6980(01)00108-3

[45] Choi OH, Kim JH, Kinet JP (1996) Calcium mobilization via sphingosine kinase in signalling by the Fc epsilon RI antigen receptor. Nature 380: 634–636. 8602265 10.1038/380634a0

[46] Tasseva G, Bai HD, Davidescu M, Haromy A, Michelakis E, et al (2013) Phosphatidylethanolamine deficiency in Mammalian mitochondria impairs oxidative phosphorylation and alters mitochondrial morphology. J Biol Chem 288: 4158–4173. 23250747 10.1074/jbc.M112.434183 PMC3567666

[47] Krebs CE, Karkheiran S, Powell JC, Cao M, Makarov V, et al (2013) The Sac1 domain of SYNJ1 identified mutated in a family with early-onset progressive Parkinsonism with generalized seizures. Hum Mutat 34: 1200–1207. 23804563 10.1002/humu.22372 PMC3790461

[48] Quadri M, Fang M, Picillo M, Olgiati S, Breedveld GJ, et al (2013) Mutation in the SYNJ1 gene associated with autosomal recessive, early-onset Parkinsonism. Hum Mutat 34: 1208–1215. 23804577 10.1002/humu.22373

[49] Stubblefield JJ, Terrien J, Green CB (2012) Nocturnin: at the crossroads of clocks and metabolism. Trends Endocrinol Metab 23: 326–333. 22608110 10.1016/j.tem.2012.03.007 PMC3389576

[50] Tain LS, Chowdhury RB, Tao RN, Plun-Favreau H, Moisoi N, et al (2009) Drosophila HtrA2 is dispensable for apoptosis but acts downstream of PINK1 independently from Parkin. Cell Death Differ 16: 1118–1125. 19282869 10.1038/cdd.2009.23 PMC2711053

[51] Dietzl G, Chen D, Schnorrer F, Su KC, Barinova Y, et al (2007) A genome-wide transgenic RNAi library for conditional gene inactivation in Drosophila. Nature 448: 151–156. 17625558 10.1038/nature05954

[52] Birch-Machin MA, Briggs HL, Saborido AA, Bindoff LA, Turnbull DM (1994) An evaluation of the measurement of the activities of complexes I-IV in the respiratory chain of human skeletal muscle mitochondria. Biochem Med Metab Biol 51: 35–42. 8192914 10.1006/bmmb.1994.1004

