# Supplementary figures and images for: The Complex I Subunit NDUFA10 Selectively Rescues Drosophila pink1 Mutants through a Mechanism Independent of Mitophagy
Source: PLoS Genet. 2014 Nov 20;10(11):e1004815. doi: 10.1371/journal.pgen.1004815 (PMC4238976; doi:10.1371/journal.pgen.1004815)

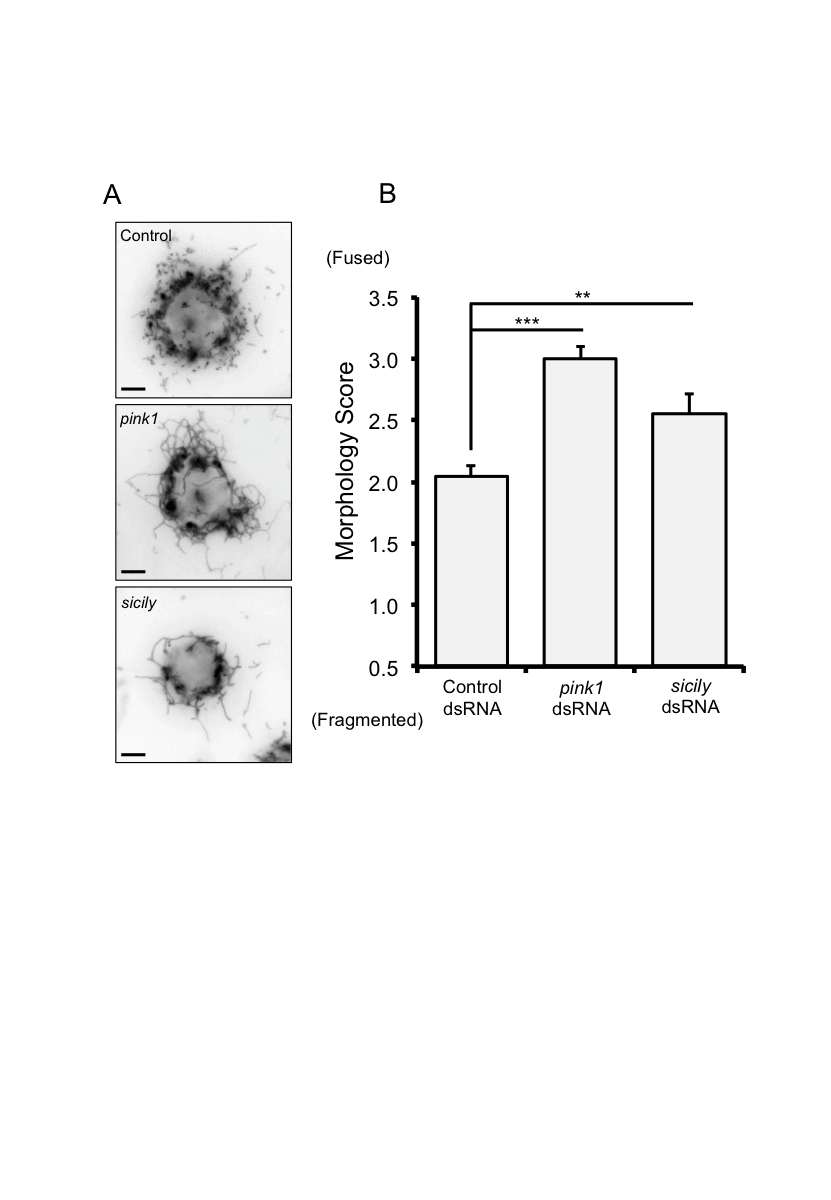

Supplement: Figure S3 — RNAi knockdown of sicily causes mitochondrial hyperfusion. (A) Drosophila S2R+ cells treated with indicated dsRNAs and stained with MitoTracker Red to visualize mitochondria. (B) Cells were scored as in Fig. 2 for relative mitochondrial morphology. Scale bar = 5 µm. ** P<0.01, *** P<0.001, Student's t-test compared with control dsRNA. (TIFF) [file pgen.1004815.s003.tiff]

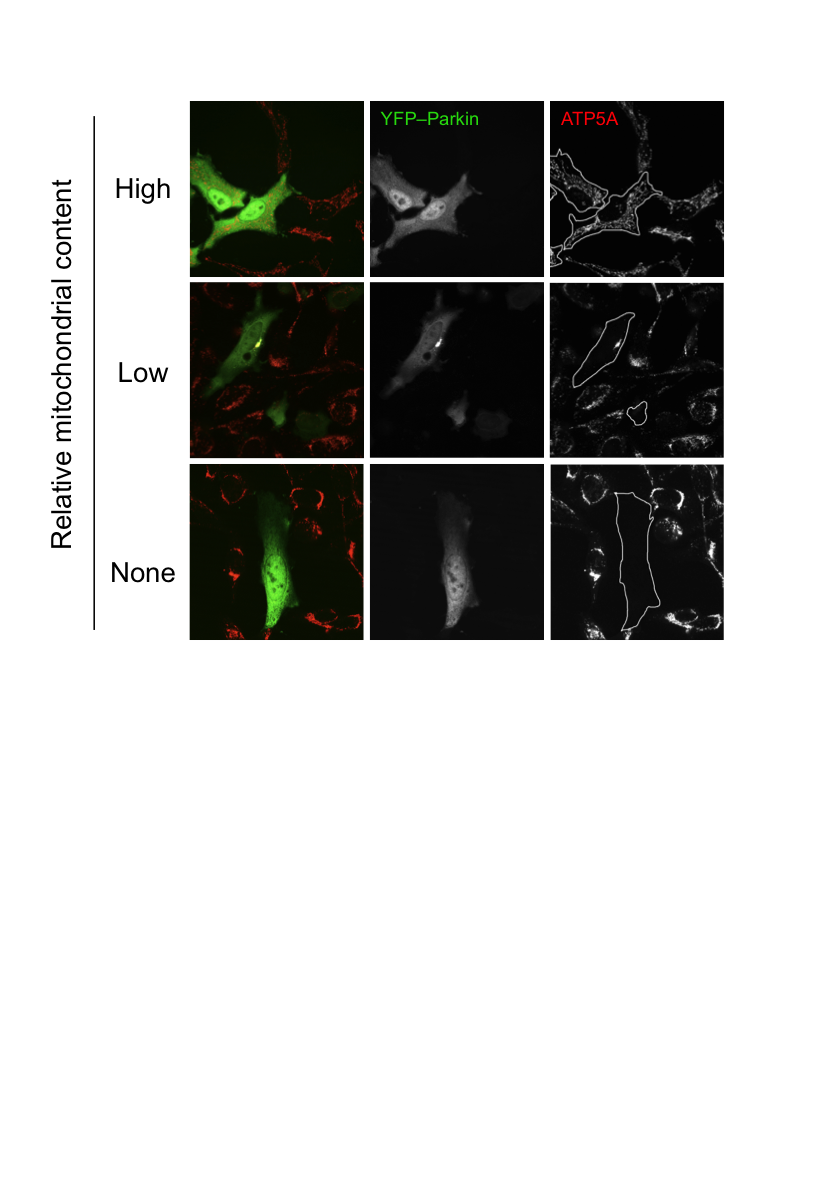

Supplement: Figure S4 — Categorization of mitochondrial content during mitophagy. HeLa cells transiently transfected to express YFP-Parkin (green) induce mitophagy following prolonged exposure to CCCP. Mitochondrial content can be monitored by ATP5A immunostaining (red). Cells with normal mitochondrial content, as seen before toxification, are categorized as “High”. Depolarized mitochondria become aggregated and in a perinuclear region, and termed “Low”. Cells which have undergone complete mitophagy have a mitochondrial content scored as “None”. (TIFF) [file pgen.1004815.s004.tiff]

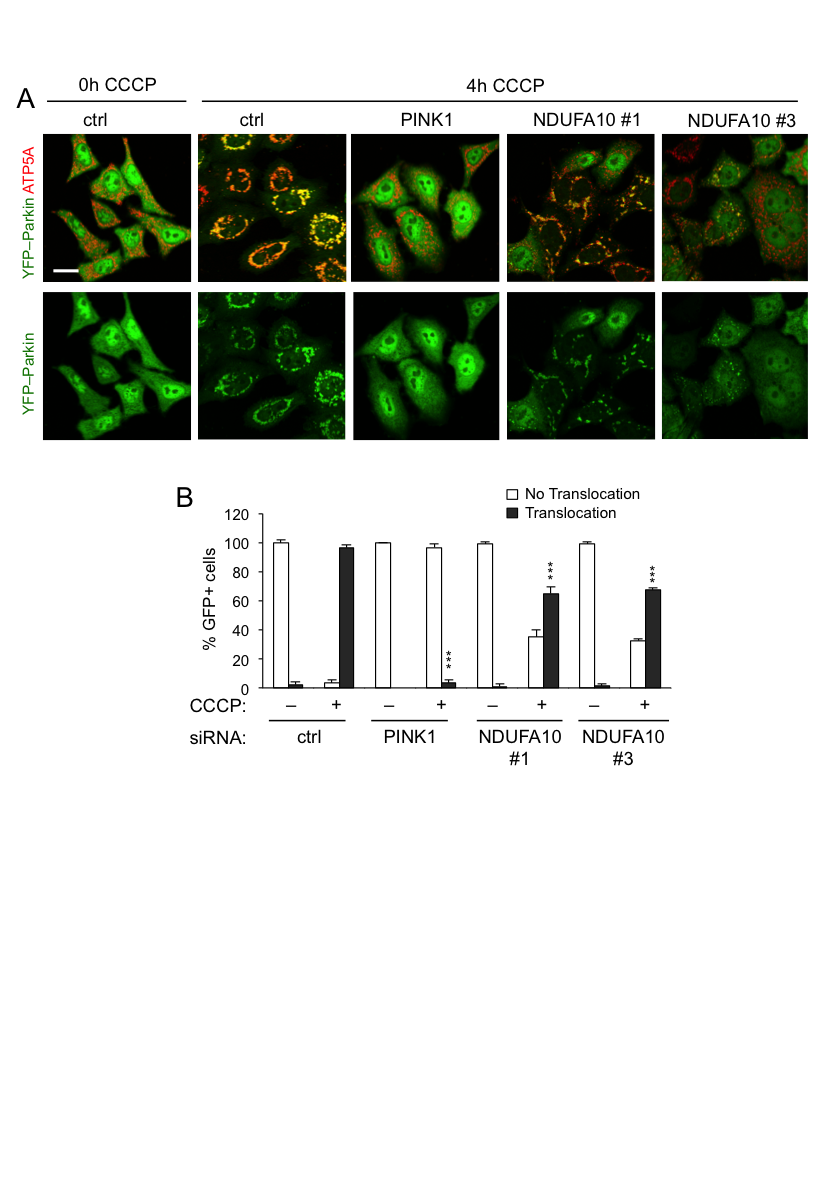

Supplement: Figure S5 — Individual NDUFA10 siRNAs attenuate CCCP-induced Parkin translocation. (A) In HeLa cells stably transfected to express YFP-Parkin, before CCCP toxification (0 h) YFP-Parkin (green) has a diffuse cytoplasmic distribution in control (ctrl) siRNA treated cells. Following 4 h CCCP, YFP-Parkin co-localizes with mitochondria labeled with ATP5A immunostaining (red). PINK1 siRNA treatment almost completely abolishes YFP-Parkin translocation. Individual NDUFA10 siRNAs, #1 and #3, significantly reduce YFP-Parkin translocation. (B) Quantification of YFP-Parkin translocation as in A, scored in triplicate experiments. n>30 cells per experiment. Scale bar = 20 µm. *** P<0.001, Student's t-test compared with control siRNA. (TIFF) [file pgen.1004815.s005.tiff]

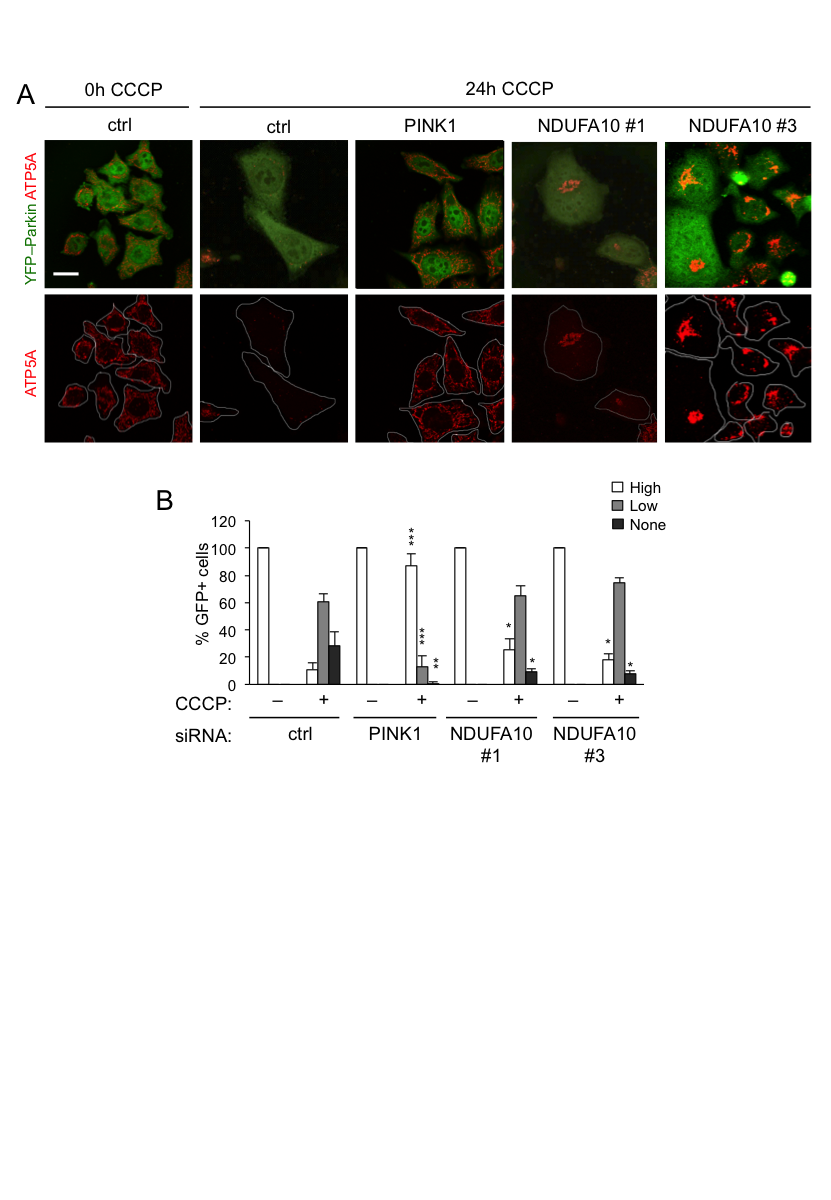

Supplement: Figure S6 — Individual NDUFA10 siRNAs reduce CCCP-induced mitophagy. (A) Stably transfected HeLa cells expressing YFP-Parkin, before CCCP treatment (0 h, ctrl) have a normal (“High”) mitochondrial content. Following 24 h treatment with CCCP, a high proportion of control cells (ctrl) show complete degradation (“none”) or perinuclear aggregated (“low”) mitochondria, visualized by ATP5A immunostaining (red). PINK1 siRNA treatment almost completely abolishes mitophagy. Individual NDUFA10 siRNAs, #1 and #3, significantly reduce mitophagy. (B) Quantification of mitochondrial content as in A, scored in triplicate experiments. n>30 cells per experiment. Scale bar = 20 µm. * P<0.05, ** P<0.01, *** P<0.001, Student's t-test compared with control siRNA. (TIFF) [file pgen.1004815.s006.tiff]

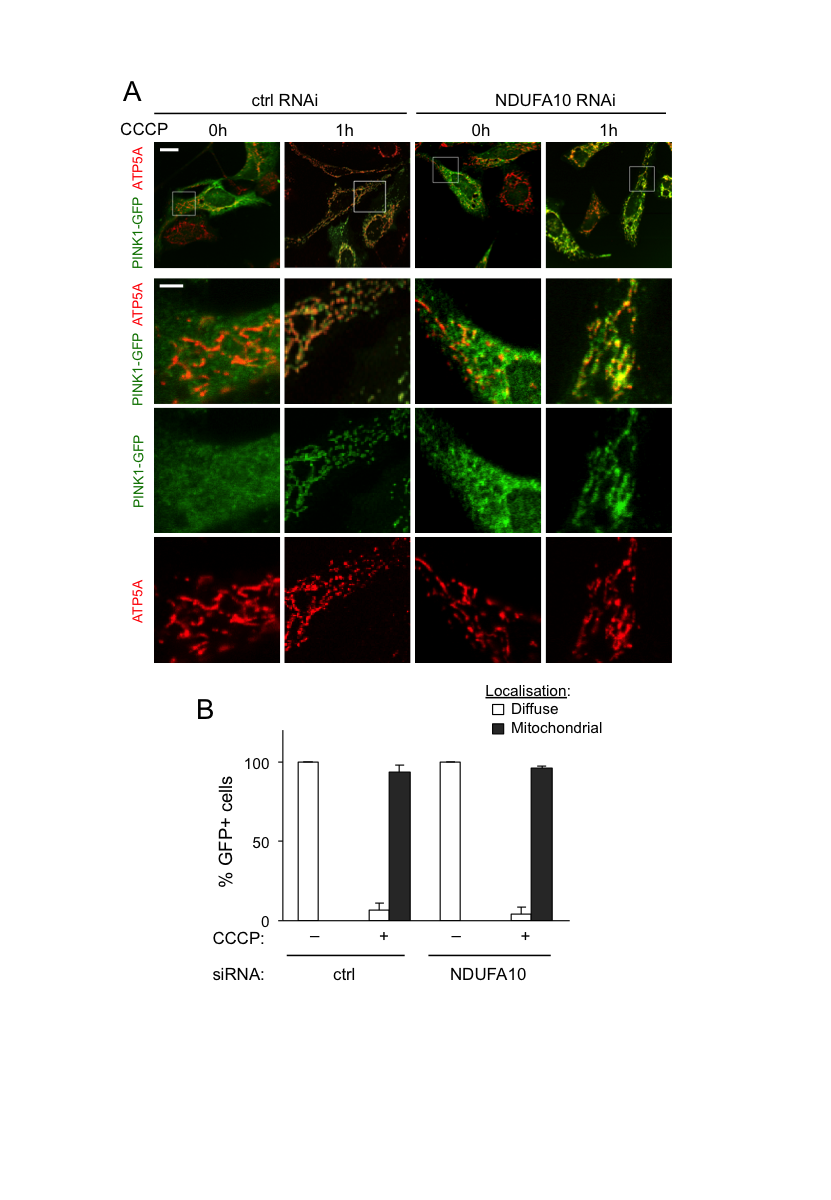

Supplement: Figure S7 — NDUFA10 knockdown does not affect CCCP-induced pink1 stabilization. (A) HeLa cells transiently expressing PINK1-GFP and treated with control (ctrl) siRNA. Before CCCP toxification (0 h) PINK1-GFP (green) has a diffuse distribution. Following 1 h CCCP PINK1-GFP becomes stabilized and accumulates on mitochondria, labeled with ATP5A immunostaining (red). Boxed areas are shown magnified in images below. (B) Quantification of PINK1-GFP stabilization as in A. Charts indicate mean ± s.d. of triplicate experiments. n>15 cells per experiment. Scale bars; low mag. = 20 µm, zoom = 4 µm. (TIFF) [file pgen.1004815.s007.tiff]
